# Machine Learning-Based Approach Highlights the Use of a Genomic Variant Profile for Precision Medicine in Ovarian Failure

**DOI:** 10.3390/jpm11070609

**Published:** 2021-06-27

**Authors:** Ismael Henarejos-Castillo, Alejandro Aleman, Begoña Martinez-Montoro, Francisco Javier Gracia-Aznárez, Patricia Sebastian-Leon, Monica Romeu, Jose Remohi, Ana Patiño-Garcia, Pedro Royo, Gorka Alkorta-Aranburu, Patricia Diaz-Gimeno

**Affiliations:** 1 IVI Foundation-Instituto de Investigación Sanitaria La Fe, Av. Fernando Abril Martorell 106, Torre A, Planta 1ª, 46026 Valencia, Spain; Ismael.henarejos@ivirma.com (I.H.-C.); alejandro.aleman@ivirma.com (A.A.); patricia.sebastian@ivirma.com (P.S.-L.); 2 Department of Paediatrics, Obstetrics and Gynaecology, University of Valencia, Av. Blasco Ibáñez 15, 46010 Valencia, Spain; remohi@ivirma.com; 3 IVI-RMA Pamplona, Reproductive Medicine, C/Sangüesa, Número 15-Planta Baja, 31003 Pamplona, Spain; begona.martinez@ivirma.com (B.M.-M.); pedro.royo@ivirma.com (P.R.); 4 CIMA Lab Diagnostics, University of Navarra, IdiSNA, Avda Pio XII, 55, 31008 Pamplona, Spain; jgraazn@unav.es (F.J.G.-A.); apatigar@unav.es (A.P.-G.); galkorta@unav.es (G.A.-A.); 5 Hospital Universitario y Politécnico La Fe, Av. Fernando Abril Martorell 106, 46026 Valencia, Spain; monicaromeuvillarroya@gmail.com; 6 IVI-RMA Valencia, Reproductive Medicine, Plaça de la Policia Local, 3, 46015 Valencia, Spain; 7 Laboratorio de Pediatría-Unidad de Genética Clínica, Clínica Universidad de Navarra, Avda Pio XII, 55, 31008 Pamplona, Spain

**Keywords:** ovarian failure, whole exome sequencing, single nucleotide variant, infertility, precision medicine, prediction model, genomic taxonomy, genome variant profile, personalized medicine, ovary

## Abstract

Ovarian failure (OF) is a common cause of infertility usually diagnosed as idiopathic, with genetic causes accounting for 10–25% of cases. Whole-exome sequencing (WES) may enable identifying contributing genes and variant profiles to stratify the population into subtypes of OF. This study sought to identify a blood-based gene variant profile using accumulation of rare variants to promote precision medicine in fertility preservation programs. A case–control (*n* = 118, *n* = 32, respectively) WES study was performed in which only non-synonymous rare variants <5% minor allele frequency (MAF; in the IGSR) and coverage ≥ 100× were considered. A profile of 66 variants of uncertain significance was used for training an unsupervised machine learning model to separate cases from controls (97.2% sensitivity, 99.2% specificity) and stratify the population into two subtypes of OF (A and B) (93.31% sensitivity, 96.67% specificity). Model testing within the IGSR female population predicted 0.5% of women as subtype A and 2.4% as subtype B. This is the first study linking OF to the accumulation of rare variants and generates a new potential taxonomy supporting application of this approach for precision medicine in fertility preservation.

## 1. Introduction

Ovarian failure (OF) is characterised by accelerated attrition of the ovarian follicle reserve, amenorrhea, dramatic hypoestrogenism, and elevated gonadotropin levels, but these manifestations differ depending on aetiology [1,2,3]. OF may result from genetic (familial or sporadic), cytogenetic, environmental, iatrogenic, autoimmune, or metabolic disorders; genetic causes account for about 10–25% of cases [4,5], and autoimmune conditions account for 4–30% of cases [6]. Though women with OF can achieve pregnancy [7,8], OF usually presents as infertility because the ovarian reserve is nearly or completely exhausted [9]. OF is often diagnosed as idiopathic, and research is needed to better define the origins of OF and identify risk factors to aid in early diagnosis and inform treatment measures [10,11,12].

Anti-Müllerian hormone (AMH) and follicle stimulating hormone (FSH) measurements aid OF diagnosis [13,14]. However, both are limited as predictive biomarkers because modest increases or decreases in AMH are difficult to detect and do not characterise subtypes of OF; meanwhile, FSH has less sensitivity than AMH and depends on the day of the cycle in which the sample is obtained [15,16,17]. Intriguingly, as for other conditions [18], high-throughput genomics data may help discern subtypes and stages of OF.

Heritability plays a clear role in OF, and studies of familial OF had shed light on genetic aspects of the condition [19,20,21]. While familial studies focus on detecting causative variants in one or few genes in one family, population studies identify variants shared by individuals independent of familial relationship and inheritance [22,23,24]. Next-generation sequencing (NSG) based on whole-exome sequencing (WES) characterises known and unknown variation within gene-coding regions in each studied sample, significantly improving the power of previous studies focused on discovery of variants in the population [19,24,25,26,27].

Despite advantages of WES, the large degree of genetic variation creates challenges in identifying meaningful changes. Therefore, strategies are needed to identify candidate variants that can be prioritised by predicted protein defects, frequency in the population, and evidence of evolutionary pressure including negative selection [22,28,29,30,31,32]. Existing studies often lack (1) negative controls (i.e., age-matched individuals without OF); (2) characterisation of genetic variation identified in genes not known to be associated with the phenotype of interest; and (3) implementation of machine learning strategies such as machine learning algorithms that provide a more comprehensive picture, as is required in reproductive precision medicine [18]. Manipulation of this information by machine learning algorithms, can be applied to stratify populations into disease subtypes based on an additive model considering the presence or absence of DNA variants [33,34,35]. Thus, we performed a WES-based case–control study to describe genomic profiles based on multivariant models considering the presence or absence of DNA variants (single-nucleotide variants (SNVs)) as preventive screening to identify women at risk of OF. This predictive model will inform precision medicine in fertility preservation programs.

## 2. Materials and Methods

### 2.1. Participants and Inclusion Criteria

A WES-based case–control study was conducted between 2017 and 2019 at infertility clinics in collaboration with a genetic diagnosis and reproductive medicine research department. The study recruited 118 women diagnosed with OF and 32 women as controls from Spain. Women exhibiting amenorrhea for >6 months with AMH values < 0.3 ng/mL, FSH values > 20 IU, and <5 follicles upon antral follicle count (AFC) via transvaginal ultrasound were classified as OF. Controls had AMH values > 1.5 ng/mL, FSH < 10 IU, and AFC > 10. Clinical outcome (live birth (LB)) for both groups was determined. All patients were <40 years old at the time of recruitment and were selected following clinical criteria with idiopathic disease and normal karyotypes, no *FRM1* permutations, and no history of pelvic surgery, radiotherapy, chemotherapy, or autoimmune disorders. The International Genome Sample Resource (IGSR) database, which contains genomic variant information including allele frequencies, normal genomic variability, and ethnicity from 1271 healthy female individuals (OF was not considered an exclusion criterion), was used as a pseudo-control population to optimise the study complementing control population [36,37,38]. The Shapiro–Wilk test was used to check normality of clinical variables, hormone levels, age, body mass index, AFC, and LB, while the Wilcoxon and Fisher tests were used to evaluate clinical ranges of AMH and presence or absence of variants, respectively, between cases and controls [39,40,41]. The study was approved by the institutional review board of the Instituto Valenciano de Infertilidad and Hospital La Fe (1709-PAM-090-PR).

### 2.2. Pre-Processing, NGS, and Variant Calling

Peripheral blood genomic DNA was isolated (Maxwell 16 lev blood DNA, Promega, Madison, WI, USA) and quantified using fluorescence spectroscopy (Qubit). Absorbance readings with Nanodrop confirmed the purity of DNA, with all samples yielding a 260:280 ratio of >1.8. DNA integrity was evaluated using TapeStation (Agilent Technologies, Santa Clara, CA, USA), and the DNA integrity number (DIN) was >7 (recommended threshold for NGS library prep strategies) for each sample. WES was performed on all DNA samples using SureSelect Clinical Research Exome V2 (Agilent Technologies) and Illumina sequencers (MiSeq or NextSeq). Reads from the 18,311 sequenced genes were aligned to the human reference genome (hg19) using the Burrows-Wheeler algorithm (version 0.7.17) mapper [42]. Subsequent variant calling was performed using GATK software (version 3.6.0) following the standard pipeline the standard pipeline recommended by the developers of the software [43]. Variants were annotated using SnpEff software (version 4.3) [44] to obtain information on which position of the genome is affected by the variant, including if it is a protein coding sequence, which gene and in what position of said gene is located, and the biological consequences expected from the changes (e.g., if the variant disrupts the triplet reading frame of the DNA, is called a Frameshift variant). Furthermore, SnpEff retrieves information from the IGSR that allow the user to know if a certain variant is registered or not in the database [44].

### 2.3. Variant Filtering, Processing, and Prioritisation

Variants were filtered based on several criteria (further detailed in Supplemental Figure S1):

1. A moderate or deleterious effect on protein coding sequence (according to SnpEff annotations). Moderate effect included Missense variants, UTR (5′ + 3′) and splice (acceptor or donor) variants; while deleterious effects included Frameshift, Nosense (stop codon gain/loss) variants, protein to protein contact modifier variants, structural interaction modifier variants and disruptive inframe variants.
2. Variants absent from the IGSR database were retained for downstream analysis. Meanwhile, variants present in the IGSR were only kept if their minor allele frequency (MAF) was lower than <0.05, based on the premise that purifying selection decreases allele frequency of variants that confer less fitness.
3. Passage of quality criteria for coverage (>100×) as well as several parameters evaluated by GATK: Genotype Quality (GQ), which evaluates the confidence of the genotype attributed to a patient for a certain variant (homozygous for the reference allele, heterozygous or homozygous for the disease-associated allele); Position depth (DP) or total number of reads detected at a given position of the genome; Allele Depth (AD), the number of reads for the variant in that position. Further information is annexed with Supplementary Figure S1.

After applying these filters, remaining variants were used to (i) identify variants with significant differences in frequency between controls and cases and (ii) classify variants if they were present in at least 10% of cases and completely absent from controls. This last point was based on the premise that variants found only in one individual are related to individual variation while variants shared by a subgroup have a higher probability of being biomarkers related to the disease. Prioritized variants in this step were researched to find possible links with fertility. In addition to IGSR, GnomAD, and dbSNP databases were consulted to determine if identified variants were already reported [45,46], and Genecards, Uniprot, and Gene Ontology databases were used to annotate gene function [47,48,49]. Case and control variant frequencies were tested by Fisher test in the R environment (version 3.4.4, 15 March 2018) [50], while variant processing and prioritisation were done in the Python environment (version 3.5.2, 26 July 2016) [51].

### 2.4. Patient Stratification and Ovarian Failure Subtypes Prediction

Prioritised variants were used to stratify the study population based on patient genotype (homozygous for the reference allele, heterozygous or homozygous for the disease-associated allele). In order to find different subtypes of ovarian failure in the study population, unsupervised hierarchical clustering—with similarity and genomic distance values calculated with the Jaccard coefficient [52]—was used to group patients based on genomic variant profile. Jaccard’s formula is J (X, Y) = |X∩Y|/|X∪Y| where X is the genotype profile of the variants of one patient, while Y is the same for another patient. Calculating the similarity coefficient with Jaccard allow grouping together patients that share not only the same variants but also the same genotype for said variants. Optimal clustering was achieved with Ball and Hartigan indexes implemented in Nbclust (version 3.0) in R [53], which increase differences inter-cluster while trying to maximize similarities intra-cluster; this is, trying to find clusters of patients that are really similar by the genomic profile while separating them as much as possible from other patients that do not share similar profiles. Using the subtypes of ovarian failure generated and to generate a model capable of distinguish patients with OF, a Random Forest algorithm was trained with a 10-fold cross-validation (CV) 100 times in WEKA platform software (version 3.8.2, 22 December 2017) [54], WEKA by default stratified the folds maintaining the proportion of cases and controls (79%/21%). With Random Forest, the model created 500 forest each iteration and selected the best consensual tree [55,56]. Random forest also assigned a score to each variant based on the mean decrease in impurity (MDI), which represents how informative each variant is for stratifying the population according to genotype of said variant [57]. Key variants in the stratification were analysed to look for potential relations with fertility. Finally, to ascertain if the genomic variant profile found in our population is detected in an independent population and ensure that a determined subtype is reproducible, the female population from the IGSR database (*n* = 1271) [36] was evaluated using our predictive model.

## 3. Results

### 3.1. Clinical Characterisation and Sequencing Quality of the Study Population

**Figure 1:**
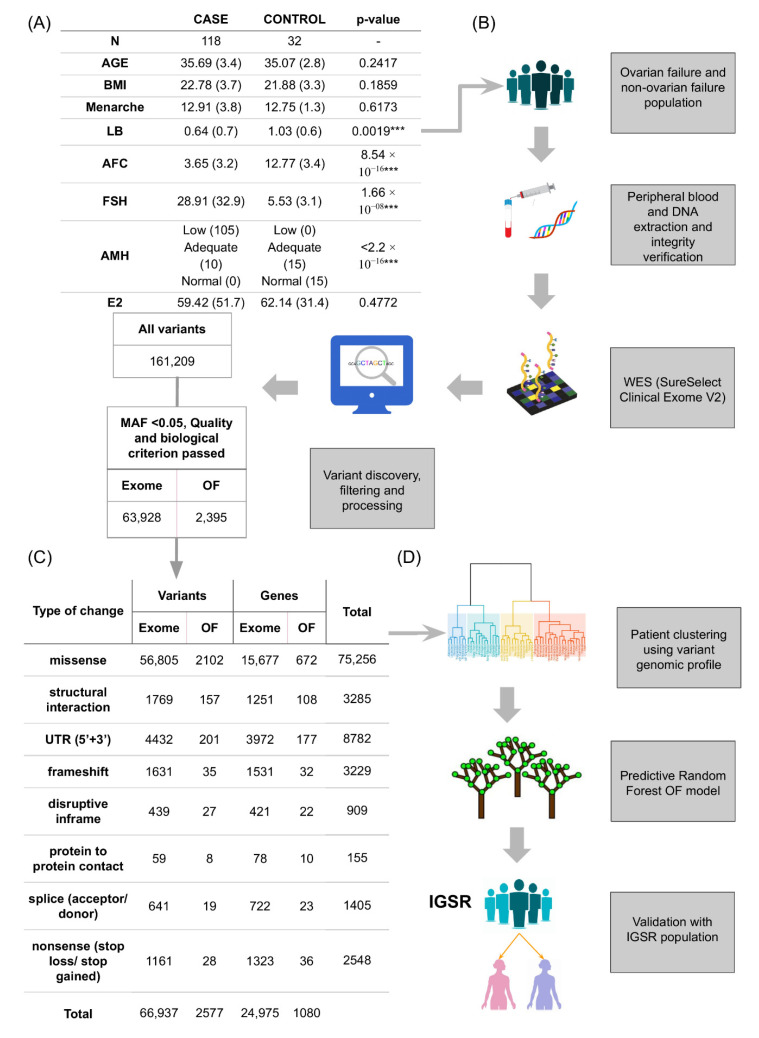
Study design and variant prioritisation. (**A**) Population demographics and clinical data. Means and standard deviations (in brackets) are shown. For age, body mass index (BMI), age at menarche, live birth (LB), antral follicle count (AFC), follicle stimulating hormone (FSH), and oestradiol (E2) contrasts. The Shapiro–Wilk test was used to check normality and the Wilcoxon test to evaluate differences between cases and controls. The Fisher test was used to evaluate differences between cases and controls for anti-Müllerian hormone (AMH) levels according to Reference Laboratory hormonal ranges (low: <0.68 ng/mL; adequate: 0.68–2.27 ng/mL; normal: >2.27 ng/mL) (*** *p* < 0.01). (**B**) Pipeline for filtering variants. Women diagnosed with ovarian failure (OF) were recruited as cases (*n* = 118) and those without ovarian failure as controls (*n* = 32). Whole-exome sequencing (WES) of DNA from peripheral blood was performed in all samples using SureSelect Clinical Research Exome V2 (Agilent Technologies) and Illumina sequencing (Miseq or Nextseq). Variant calling was performed using GATK software. (**C**) Prioritised variants. Variants that passed quality and biological criteria (minor allele frequency (MAF), type of change at protein level, sequencing parameters) are shown for targeted analysis of genes previously associated with ovarian physiology and from whole exome analysis. Number of variants and genes affected for each predicted change in protein function are also represented. (**D**) Pipeline of the predictive OF model. A random forest predictive model is built using the prioritised variants and then validated with the pseudo-control population of the International Genome Sample Resource (IGSR) (*n* = 1271).

High-depth exome sequencing data were achieved with an average > 100× for 15,903 genes (86.85% of the studied 18,311 genes) and >25× for 16,773 genes (91.66% of the studied 16,773 genes) among 118 patients with OF and 32 controls. Phenotypically characterised cases and controls had significantly different mean values of AMH, AFC, FSH, and LB (*p* < 0.01), highlighting the clinical differences associated with OF related to controls (Figure 1A). For genomic study of SNVs associated with OF, we focused on variants absent in the IGSR with a low MAF (<0.05) in the IGSR population and changes predicted to cause a moderate to deleterious effect at the protein level. With these criteria, we ensured that variants were under pressure of purifying selection.

### 3.2. Genomic Variation Hypotheses

Gene-targeted and non-targeted hypotheses related to ovarian failure were developed from the 161,209 variants identified in the 18,311-gene panel (Figure 1B). In the gene-targeted approach, we focused on finding more variants in genes previously associated with ovarian physiology; for the non-targeted approach, the whole gene panel was considered to identify main variants in novel genes not previously associated with OF. There were 2395 variants in genes associated with ovarian physiology identified in the targeted approach, and 63,928 variants considered candidate disease-associated alleles in the non-targeted approach (Figure 1C); 57,866 synonymous variants were excluded because no effect was predicted. However, contrasting the proportions of variants found in targeted genes to the proportions of variants found in remaining genes of the exome (non-targeted) revealed that targeted genes related to ovarian physiology accumulated fewer variants (Fisher test, *p* < 2.2 × 10^−16^, odds ratio = 1.2), so the targeted hypothesis was discarded. Variants found in the whole exome were classified by the predicted type of change in the coding sequence. The 63,928 variants showed moderate to deleterious effects, with most changes being missense in the UTR or in structural interaction and frameshifts (Figure 1C). The rest of the experimental design covered in the following points is shown in Figure 1D.

### 3.3. A Genomic Variant Profile Predictive of OF

A significant difference (*p* < 0.01) in the distributions of allele frequencies between cases and controls was observed in 116 of the 63,928 candidate variants (Supplementary Table S1). Interestingly, only one of them, the missense variant c.902C > G (p.Ala301Gly) inducing an alanine to glycine change, was identified in the macrophage stimulating 1-like *(MST1L*) gene in 14 controls and four cases with a significant difference in proportions (FDR = 0.03, odds ratio = 16.6) (highlighted in Supplementary Table S1). Given the unique intra-variability of each individual and that finding variants shared by several individuals is complex, to ensure that the accumulation of variants was predictive of OF, we identified variants shared by at least 10% of cases and not present in controls. The 66 variants of uncertain significance (VUS) absent in controls with high case prevalence in >10% of cases affected 62 genes (Supplementary Table S2). Important variants by their prevalence in cases are shown in Table 1. One variant, affecting the mucin 6 (*MUC6)* gene (c.5297C > T; p.Thr1766Ile), was identified in 26% (i.e., 31 of 118) of patients with OF. An additional variant, c.715G > A (p.Ala239Thr), affecting the ankyrin repeat domain 20 family member A4 (*ANKRD20A4*), was absent in IGSR, dbSNP, and GnomAD databases. In addition to the *MUC6* variant, five variants were shared by >20 cases: c.529A > G (p.Ser177Gly) affecting bromodomain and PHD finger containing 3 (*BRPF3)* in 22 cases, c.1435G > A (p.Ala479Thr) affecting adaptor related protein complex 5 subunit mu 1 (*AP5M1*) in 22 cases, c.880A > T (p.Met294Leu) affecting cysteine rich secretory protein LCCL domain containing 2 (*CRISPLD2)* in 21 cases, c.692C > G (p.Ala237Gly) affecting galactosamine (N-acetyl)-6-sulfatase (*GALNS)* in 20 cases, and c.539C > G (p.Thr180Ser) affecting mini chromosome maintenance complex component 5 (*MCM5)* in 20 cases (highlighted in Table 1). Three of the 66 variants affected three genes previously associated with infertility: variant c.181G > C (p.Ala60Pro) of mutS homolog 3 (*MSH3)* in 15 cases, c.1534G > A (p.Val512Ile) in gamma-glutamyltransferase 1 (*GGT1)* in 14 cases, and c.782G > A (p.Arg261Gln) of aquaporin 8 (*AQP8*) in 13 cases (as noted in Table 1).

### 3.4. A New Genomic Taxonomy of OF

**Figure 2:**
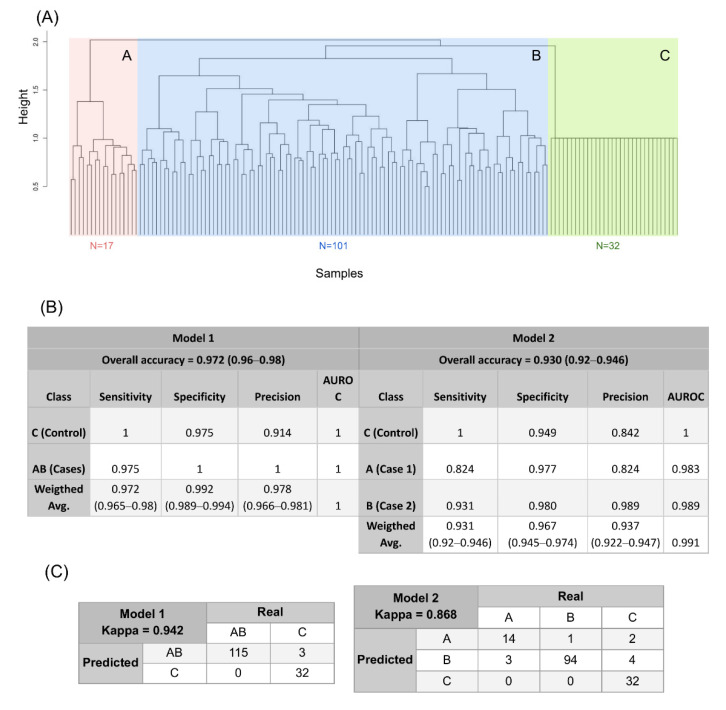
Genomic taxonomy of ovarian failure. (**A**) Dendrogram obtained from unsupervised clustering of case and control individuals. Three clusters are distinguished: clusters A (red, *n* = 17) and B (blue, *n* = 101) group case individuals into two distinct genomic profiles, while cluster C (green, *n* = 32) contains all control individuals. Genomic distance is represented by height for all groups, with a greater height indicating a larger difference between groups. (**B**) Prediction performance parameters. Parameters were obtained after executing a random forest algorithm 100 times, with 500 trees created in each iteration with 10-fold stratified cross-validation. Parameters are shown for model 1 (**left**) and model 2 (**right**), with the corresponding values of accuracy, sensitivity, specificity, precision, and ROC area obtained for each class and a weighted average in total. Kappa statistic for each model also is shown. (**C**) Prediction performance confusion matrices. A matrix is shown for model 1 (**left**), where controls (group C) were distinguished from cases (groups A, B). All 32 controls were correctly classified in cluster C, but three cases were misclassified. A matrix for model 2 (**right**) distinguishing the two genomic profiles for ovarian failure (groups A and B), with all controls correctly classified (group C) and 10 cases incorrectly classified as either controls or the other subtype. AUROC = area under ROC curve.

**Figure 3:**
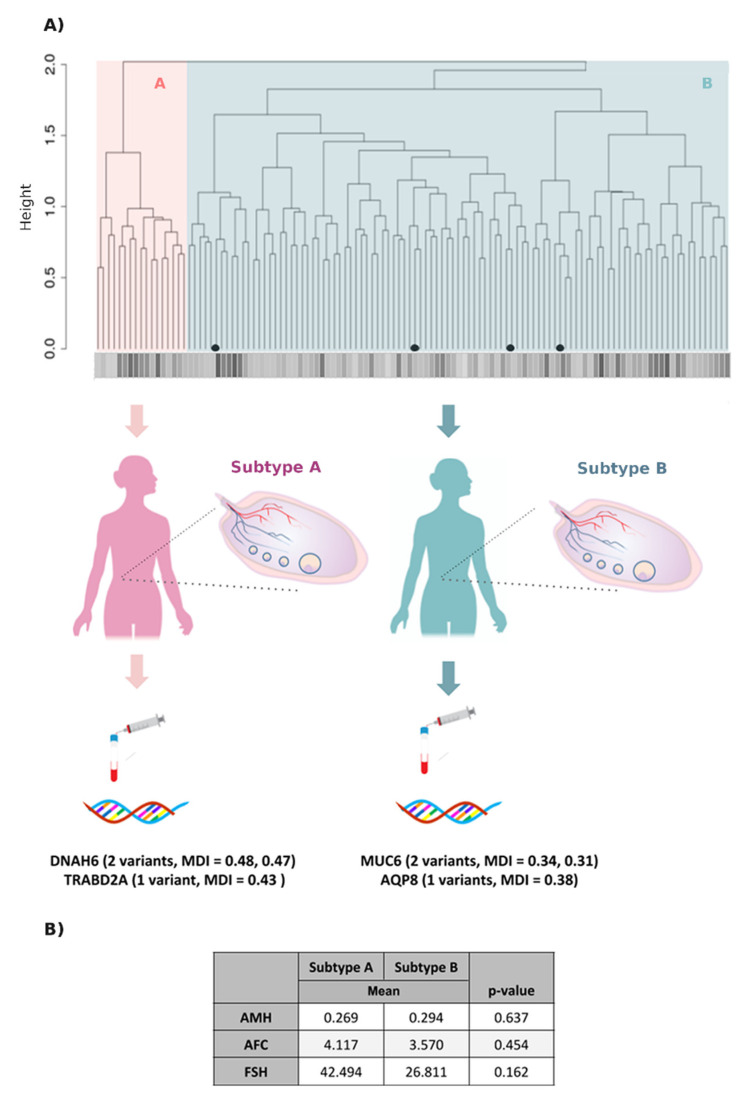
Characterisation of the two genomic subtypes of ovarian failure. (**A**) Dendrogram analysis of both subtypes. Genomic profiles of subtypes A (red cluster, *n* = 17) and B (blue cluster, *n* = 101) obtained from unsupervised clustering were examined for the number of variants accumulated in each individual (represented in greyscale). Macrophage stimulating 1 like variant was found in four cases, all in the B subtype (black point in dendrogram). Mean decrease in impurity (MDI) scores assigned by the random forest algorithm was evaluated, and two genomic variants for dynein axonemal heavy chain 6 (*DNAH6*) and one for TraB domain containing 2A (*TRABD2A*) genes were the most characteristic of subtype A. Meanwhile, two variants for mucin 6 (*MUC6*) and one for aquaporin 8 (*AQP8*) were the most characteristic of subtype B (bottom). (**B**) Clinical comparison of subtypes A and B. Shapiro and Wilcoxon tests were applied to contrast the mean antral follicle counts (AFC) and levels of follicle-stimulating hormone (FSH) and anti-Müllerian hormone (AMH). No statistical differences were found.

Based on the 66 variants present in >10% of OF cases and absent in controls, the clustering based on the genomic variant profile distinguished two main subtypes of OF (subtypes A and B) distinct from controls (C) (Figure 2A). Based on genomic distance, subtype B was more similar to controls than A. The predictive value of the 66 variant profiles distinguished OF cases (A, B) and controls (C) with an average of 97.2% (ranging 0.96–0.98) through the 100 interactions of the Random Forest model, an average sensitivity of 97.2% (ranging 0.965–0.98) an average specificity of 99.2% (ranging 0.989–0.994) (Figure 2B, left). Major variant contributors to the stratification were *SPEP1* and *GAB4* missense variants (c.1369C > A, p.Arg457Ser and c.818T > C, p.Leu273Pro, with MDI 0.2 and 0.15, respectively). The model also distinguished two genomic subtypes of OF (A and B) with an average of 93.3% accuracy (ranging 0.92–0.946) through the 100 iterations of the model, an average of 93.31% (ranging 0.92–0.946) sensitivity, and an average of 96.57% specificity (ranging 0.945–0.974) (Figure 2B, right); 14.4% of OF patients were classified as type A and 85.6% as type B, and three patients were incorrectly classified between cases and controls (Figure 2C, left) and an average of 10 patients were incorrectly classified as the other subtype or control when comparing subtypes (Figure 2C, right). Further, there were no clinical differences of significance in mean values for AMH, FSH, or AFC between subtypes A and B, so the difference was only at the genetic level (Figure 3B). The number of disease-associated variants (*n* = 66) accumulated by each patient ranged 1–15, with most accumulating nine variants (*n* = 17) (Supplementary Figures S2 and S3A). Additionally, the *MST1L* variant c.902C > G (p.Ala301Gly) was confined to subtype B (highlighted in Figure 3A). Genomic characterisation of the subtypes revealed three variants characteristic of subtype A; two affecting the dynein axonemal heavy chain 6 *(DNAH6)* gene (c.6356A > G, p.Tyr2119Cys and c.8576A > G, p.Lys2859Arg, MDI 0.48 and 0.47, respectively), and one affecting traB domain containing 2A *(TRABD2A*) (c.1034G > A, p.Arg345His, MDI 0.43); also identified were two previously mentioned variants (*AQP8* and *MUC6* (MDI 0.38 and 0.31, respectively)) and a second *MUC6* variant (c.5330G > A, p.Gly1777Asp, MDI 0.34) characteristic of subtype B (Figure 3A, bottom) (as noted in Table 1).

### 3.5. Testing the Genomic Predictive Model in the IGSR Population

To determine whether the model could predict subtypes of OF in an independent population with unknown fertility, we tested our model in the female IGSR population (*n* = 1271). A prediction score of 0–1 was associated with each individual to determine diagnostic power, and only individuals with a predictive score ≥ 0.9 were considered predicted to the specific subtype. In the IGSR population, 7/1271 (0.5%) women were predicted as subtype A, while 31/1271 (2.4%) were considered subtype B.

## 4. Discussion

We describe the first predictive model of OF based on a genomic variant profile obtained through blood WES and machine learning algorithms. The model was effective in identifying and stratifying patients into two subtypes of OF (A and B), considering a pattern of 66 variants rather than individual variant effects. Subtypes of OF were tested in an independent IGSR population of 1271 women—only 0.5% of women were predicted as subtype A, a feasible proportion considering that OF prevalence is estimated at 1% in women < 40 years old [1,2,3]. We believe that the 2.4% of women predicted as subtype B may be overestimated given that our model was constructed under the assumption that the 66 variants are absent from controls and our design only considered 32 controls. However, the IGSR population introduced a higher population variability that could contribute to false positives in subtype B, as this profile was more similar to controls than subtype A. Additionally, other phenomena influencing disease prevalence such as penetrance or expressivity together with environmental factors could affect the final phenotype. Thus, subtype A should be considered the most distinguishable from controls, with likely fewer false positives and with the most potential to be useful in fertility preservation programs. However, further prospective studies are needed to evaluate the prediction ability of this model in relation to clinical phenotype.

Prior exome-sequencing studies seeking to identify new variants associated with OF [22,30,58] focused on established genes associated with OF; in our study, DNA sequences in genes related to ovarian physiology exhibited proportionately fewer variants than remaining genes in the exome. Further, prior studies identified variants shared by a few patients with OF and did not include controls [22,29,31,32,59,60]. The prioritisation criteria used in our study ensure that variants are rare and are likely under purifying selective pressure based on the potential cumulative adverse effects of the variants on genetic fitness of the OF population at a functional level [25,61]. In contrast to GWAS studies that use populations of thousands [62,63,64,65], our study had a modest sample size but was larger than similar studies in Europe or the USA [22,32,66,67]. The efficacy of the contrast of proportions approach was lower than GWAS studies, as expected, although we did identify 116 disease-associated variants with *p* < 0.01 and one variant with an adjusted *p* < 0.05 that was over-represented in controls. This adjusted variant affected *MST1L*, which encodes a protein with serine-type endopeptidase activity but no other known functions [47,48]. Overrepresentation of the *MSTL1* variant in controls may protect against OF, suggesting an advantage conferred by the G allele, but this requires further research because the number of controls in this study was small.

The fingerprint or genomic intra-variability of each individual presents a challenge in variant profiling [68]. A presence > 10% (i.e., 12 out of 118 OF patients) was deemed necessary to identify variants fixed in the OF population, and we identified 66 VUS in genes not previously associated with OF matching these criteria. In addition to identifying variants in genes not previously associated with fertility, we identified three variants affecting genes previously associated with OF: *MSH3*, *GGT1*, and *AQP8*. *MSH3,* part of the post-replication DNA repair system, is required for fertility; mice lacking *Mshl3* are sterile and their oocytes fail to complete meiosis I [69]. *Ggt1* knock-out mice are infertile, lack antral follicle development, and do not respond to external gonadotropins [70]. *AQP8* plays an important role in the apoptosis of granulosa cells, and mice lacking *Aqp8* develop mature follicles and are more fertile than wild-type mice [71]; therefore, further research is needed to determine how the *AQP8* variants impact OF.

The top six variants shared by patients with OF that were most representative of our population occurred in *MUC6*, *BRPF3*, *AP5M1, CRISPL2, GALNS,* and *MCM5*. A variant in *MUC6* was found in 31 cases (25% of study population). *MUC6* encodes a protein associated with protecting epithelial surfaces against chemical agents [72] and is related to ovarian tumours in mice [73]. Given that OF may have an environmental aetiology [11,20,74], we hypothesise that it may protect the ovary from environmental pollution and chemotoxicity and its variants may confer differential sensitivity to environmental agents. Another variant shared by 22 patients (20% of population) is within *BRPF3* and is associated with reorganisation of chromatin and acetylation of histone H3K14, which is needed for efficient activation of DNA replication [75]. Chromatin organisation and DNA replication are imperative during follicular development [19,76], suggesting a role of *BRPF3* in oocyte maturation and fertility. Further, an *AP5M1* variant was found in 22 women. AP5M1 induces apoptosis in cervical carcinoma cells [77] and may play a similar role in primordial follicle death and premature loss of ovarian reserve. Twenty-one cases had a variant in *CRISPL2*, which promotes extracellular matrix assembly. The bovine *CRISPL2* homolog is upregulated in granulosa cells in ovulatory follicles and could play an important role in human fertility [78]. *GALNS* was affected in 20 patients, participates in degradation of glycosaminoglycans, and is highly expressed in the ovary [79]. Finally, 20 patients had a variant affecting *MCM5*, which encodes a protein that is part of a molecular complex involved in DNA replication. Alterations in members of the same gene family, *MCM8* and *MCM9*, affect DNA repair and cause OF [80,81,82].

Precision medicine describes new disease stages and treatment targets based on genomic profiles [18]. A profile based on the 66 VUS identified in this study distinguished OF from controls with 97.2% accuracy and stratified the OF population into two different groups with 93% accuracy, 93.31% sensitivity, and 96.67% specificity. Three patients in subtype A and six in subtype B failed to classify as their actual subtype. Subtype B was closer to the control group and contained all four cases sharing the *MST1L* variant. These results suggest two genomic subtypes of OF, one with a specific genetic profile (A) and another (B) genetically distinct but closer to our control group. Two variants, one in *SPEP1* and the other in *GAB4*, were the most informative in case vs. control classification. Little is known, however, about the functions of both genes. Three gene variants were the most informative for classifying patients into subtype A (one in *TRABD2* affecting 18 patients, and two in *DNHA6* affecting 17 and 19 patients) and three into subtype B (*AQP8* and *MUC6* variants, and an additional *MUC6* variant affecting 13 patients). TRABD2A is a metalloprotease that acts as a negative regulator of WNT signalling [83] by cleaving WNT3A, which is needed in synergy with R-spondin2 for follicular development in mice [84]. TRABD1A also cleaves WNT5A, a protein that decreases ovulation and increases follicular atresia. WNT5A is a physiologic inhibitor of gonadotropin signalling in humans [85], and female *Wnt5a* knockout mice are subfertile [86]. *DNAH6* belongs to the dynein family of genes and encodes part of the microtubule-associated motor protein complex. Other dynein family members, *DNAH5* and *DNAH1*, are associated with infertility [87,88]. Mutations in *DNAH6* cause primary ciliary dyskinesia and Huntington’s disease, both of which are associated with infertility [89,90].

Although we purport functional roles for the genes involved in the genomic variant profile, this does not imply causation in reference to OF. Not all variants of the profile need to have a direct link with the pathology; these variants could be biomarkers without an implication for fertility. Importantly, the subtypes we described were distinguishable only by their genetic profiles and not by clinical parameters (including FSH), suggesting that they are detectable with the molecular deepness of genomic profiling. This highlights that a deeper understanding of the variant profile could change OF taxonomy and molecular classification. Indeed, this is interesting because the clinical criteria do not distinguish aetiologies or subtypes of OF. Subtype A could be easier to distinguish from controls for preventive detection in women at risk to experience infertility who can be identified only from genomic information. Whether these subtypes have different aetiologies or confer other clinical implications requires further studies.

We distinguished a genomic variant profile between OF cases and controls that revealed two subtypes of OF. While the inheritance and causative nature of the variants are not known, the distinct variant profiles serve as building blocks for a predictive model to detect subtype A in the general population and offer a promising first step toward using genomic and personalised medicine to predict OF in fertility preservation programs. We acknowledge that the good predictive value of our model depends on absence of the 66 variants for proper prediction; thus, as more individuals are tested, the performance of the model may decrease, such as overestimating the number of women with subtype B. Nonetheless, we believe this distinct genomic profile is capable of predicting OF in the general population, especially for subtype A. Clinical follow-up studies and prediction models testing in an independent population will be required to ascertain significance of the identified subtypes and overcome clinical and technical limitations of this study. Nevertheless, and given our sample size, we trust that the cross-validation models here developed avoids overfitting by using all samples in both training and testing phases.

## 5. Conclusions

We described the genomic profile of 66 VUS not previously associated with OF. The variant profile was used to create a predictive model capable of identifying OF individuals and classifying them into two genomic subtypes (A and B) with high accuracy, specificity, and sensitivity. One subtype was predicted accurately in a feasible proportion of the IGSR cohort as a surrogate of the general population. Thus, the identified variants may help establish a variant profile as a preventive biomarker in fertility preservation programs as a minimally invasive test in blood samples. Further prospective studies in an independent population are needed to determine reproducibility of the model and evaluate preventive potential of the two genetic subtypes in clinical practice.

## Figures and Tables

**Table 1:** Novel variants predictive of ovarian failure. Chromosomal and genomic positions of the variants, as well as bases and amino acid changes at the indicated number in the sequences, dbSNP IDs (if known), type of changes, amino acid class, polarity and charge changes, genes affected, number of cases affected by the variant, coverage (mean and standard deviation), and accession numbers. Variants were present in at least 10% of cases (12 women out of 118) and no controls. Gene Ontology and Genecards databases were consulted to annotate gene function. ^1^ Top 6 variants shared by >20 cases. ^2^ Top variants valued by random forest algorithm to stratify the population. ^3^ The three variants found affecting genes already related to ovarian failure in the literature. ^4^ Variant absent in International Genome Sample Resource, dbSNP, and GnomAD databases.

Chromosome and Position	Change at Sequence and Aa Level	Rs	Type of Change	Amino Acid Class, Polarity, and Charge Change	Gene	Function	N Cases Affected	Coverage	Accession Number
2; 84897501 ^2^	c.6356A > G, p.Tyr2119Cys	rs17025409	Missense variant	Aromatic polar neutral > sulfuric nonpolar neutral	*DNAH6*	Microtubule activity	17	146 (18.08)	NM_001370.1
2; 84932720 ^2^	c.8576A > G, p.Lys2859Arg	rs61750773	Missense variant	Basic polar positive > basic polar positive	*DNAH6*	Microtubule activity	19	146 (20.02)	NM_001370.1
2; 85059227 ^2^	c.1034C > T, p.Arg345His	rs61744273	Missense variant	Basic polar positive > basic aromatic polar positive-neutral	*TRABD2A*	Negative regulation of WNT signalling pathway	18	287 (42.23)	NM_001277053.1
5; 79950724 ^3^	c.181G > C, p.Ala60Pro	rs2001675	Missense variant	Aliphatic nonpolar neutral > cyclic nonpolar neutral	*MSH3*	DNA repair	15	145 (57.73)	NM_002439.4
6; 36168628 ^1^	c.529A > G, p.Ser177Gly	rs45504893	Missense variant	Hydroxylic polar neutral > aliphatic nonpolar neutral	*BRPF3*	Chromatin organisation	22	320 (44.1)	NM_015695.2
9; 69391207 ^4^	c.715G > A, p.Ala239Thr		Missense variant	Aliphatic nonpolar neutral > hydroxylic polar neutral	*ANKRD20A4*	Unknown	13	125 (8.03)	NM_001098805.1
11; 1017471 ^2^	c.5330G > A, p.Gly1777Asp		Missense variant	Aliphatic nonpolar neutral > acid acidic polar negative	*MUC6*	Cytoprotection of epithelial surfaces	13	448 (194.38)	NM_005961.2
11; 1017504 ^1,2^	c.5297C > T, p.Thr1766Ile		Missense variant	Hydroxylic polar neutral > aliphatic nonpolar neutral	*MUC6*	Cytoprotection of epithelial surfaces	31	448 (147.15)	NM_005961.2
14; 57755564 ^1^	c.1435G > A, p.Ala479Thr	rs35759976	Missense variant	Aliphatic nonpolar neutral > hydroxylic polar neutral	*AP5M1*	Apoptosis	22	179 (14.19)	NM_018229.3
16; 25239809 ^2,3^	c.782G > A, p.Arg261Gln	rs111840156	Missense variant	Basic polar positive > amide polar neutral	*AQP8*	Cellular response to cAMP	13	265 (24.69)	NM_001169.2
16; 84902483 ^1^	c.880A > T, p.Met294Leu	rs72799568	Missense variant	Sulfuric nonpolar neutral > aliphatic nonpolar neutral	*CRISPLD2*	Extracellular matrix assembly	21	247 (145.71)	NM_031476.3
16; 88902199 ^1^	c.692C > G, p.Ala237Gly	rs34745339	Structural interaction variant, missense variant	Aliphatic nonpolar neutral > aliphatic nonpolar neutral	*GALNS*	Degradation of glycosaminoglycans	20	214 (33.02)	NM_000512.4
17; 71232990 ^2^	c.1369C > A, p.Arg457Ser	rs61729639	Missense variant	Basic polar positive > hydroxylic polar neutral	*SPEP1*	Unknown	14	180 (38.16)	NM_001288771.1
22; 17450929 ^2^	c.841G > A, p.Ala281Thr	rs61741409	Missense variant	Aliphatic nonpolar neutral > hydroxylic polar neutral	*GAB4*	Unknown	14	236 (36.89)	NM_001037814.1
22; 17450952 ^2^	c.818T > C, p.Leu273Pro	rs11703655	Missense variant	Aliphatic nonpolar neutral > cyclic nonpolar neutral	*GAB4*	Unknown	14	236 (43.66)	NM_001037814.1
22; 25024326 ^3^	c.1534G > A, p.Val512Ile		Structural interaction variant, missense variant	Aliphatic nonpolar neutral > aliphatic nonpolar neutral	*GGT1*	Proteolysis	14	112 (17.48	NM_013430.2
22; 35802661 ^1^	c.539C > G, p.Thr180Ser	rs2307340	Missense variant	Hydroxylic polar neutral > hydroxylic polar neutral	*MCM5*	DNA replication initiation	20	309 (92.27)	NM_006739.3

## Supplementary Materials

The following are available online at https://www.mdpi.com/article/10.3390/jpm11070609/s1, Figure S1: Pipeline for filtering genomic variants. A first filter was made to retain variants affecting protein coding sequences. From these, the International Genome Sample Resource (IGSR) database, which contains variants of >3000 healthy individuals, was consulted. Variants found in the database with a minor allele frequency (MAF) < 0.05 were kept. Variants absent from the database also were retained. Two criteria were applied to this set of variants. Quality criteria values were based on confidence of a genotype attributed to a specific sample, or genotype quality (GQ); total amount of reads for a given position, or position depth (DP); and number of reads for the given variant detected in the sequencing, or allele depth (AD). In addition, AD should account for ≥20% of position depth. Biological criteria were based on the predicted protein-level effect of the variant, which could range from deleterious to moderate. Figure S2: Frequency of ovarian failure variants accumulated in each patient. Number of patients is presented on the *Y*-axis, and number of presenting variants from the genomic profile of 66 variants associated with ovarian failure on the *X*-axis of the histogram. Most patients (17) shared 9 variants. The maximum number of shared variants was 15, seen in 4 patients. Figure S3: *DNAH6* and *TRABD2A* variants. Screenshots are shown for 3 variant s (2 for DNAH6, 1 for TRABD2A), highlighting the genome position (left corner, in green) and the presence of the variant in all corresponding case samples in the respective gene (highlighted in the centre of the picture, in green). Table S1: Significant variants found after using contrast of proportions. Fisher test identified 116 significant variants (*p* < 0.01). Chromosome, position, change at sequence and amino acid level, type of change, genes affected, and dbSNP identifier, if known, are shown. ^1^ = Significant variant after adjusting p-values for FDR (adj. < 0.05). Table S2: Novel sixty-six variants predictive of ovarian failure. Chromosomal and genomic positions of the variants, as well as bases and amino acid changes at the indicated number in the sequences, dbSNP IDs (if known), type of changes, amino acid class, polarity and charge changes, genes affected, number of cases affected by the variant, and coverage and accession numbers. Variants were present in at least 10% of cases (12 women out of 118) and no controls. Gene Ontology and Genecards databases were consulted to annotate gene function. ^1^ Top 6 variants shared by >20 cases. ^2^ Top variants valued by random forest algorithm to stratify the population. ^3^ The three variants found affecting genes already related to ovarian failure in the literature. ^4^ Variant absent in International Genome Sample Resource, dbSNP, and GnomAD databases.
