# Supplementary material for: Machine Learning-Based Approach Highlights the Use of a Genomic Variant Profile for Precision Medicine in Ovarian Failure
Source: J Pers Med. 2021 Jun 27;11(7):609. doi: 10.3390/jpm11070609 (PMC8305607; doi:10.3390/jpm11070609)
Supplement: Supplementary file 1 [file jpm-11-00609-s001.zip › jpm-1233323-supplementary.pdf]

Machine learning-based approach highlights the use of a genomic variant profile for precision medicine in ovarian failure

Ismael Henarejos-Castillo[1,2], Alejandro Aleman[1], Begoña Martinez-Montoro[3], Francisco Javier Gracia-Aznárez[4], Patricia Sebastian-Leon[1,3,5], Monica Romeu[5], Jose Alejandro Remohi[2,6], Ana Patiño-Garcia[4,7], Pedro Royo[3], Gorka Alkorta-Aranburu[4], and Patricia Diaz-Gimeno[1,3,5]*

Supplementary information

Outline

- **Figure S1. Pipeline for filtering genomic variants.**
- **Figure S2. Frequency of ovarian failure variants accumulated in each patient.**
- **Figure S3. *DNAH6* and *TRABD2A* variants.**
- **Table S1. Significant variants found after using contrast of proportions.**
- **Table S2. Novel sixty-six variants predictive of ovarian failure.**

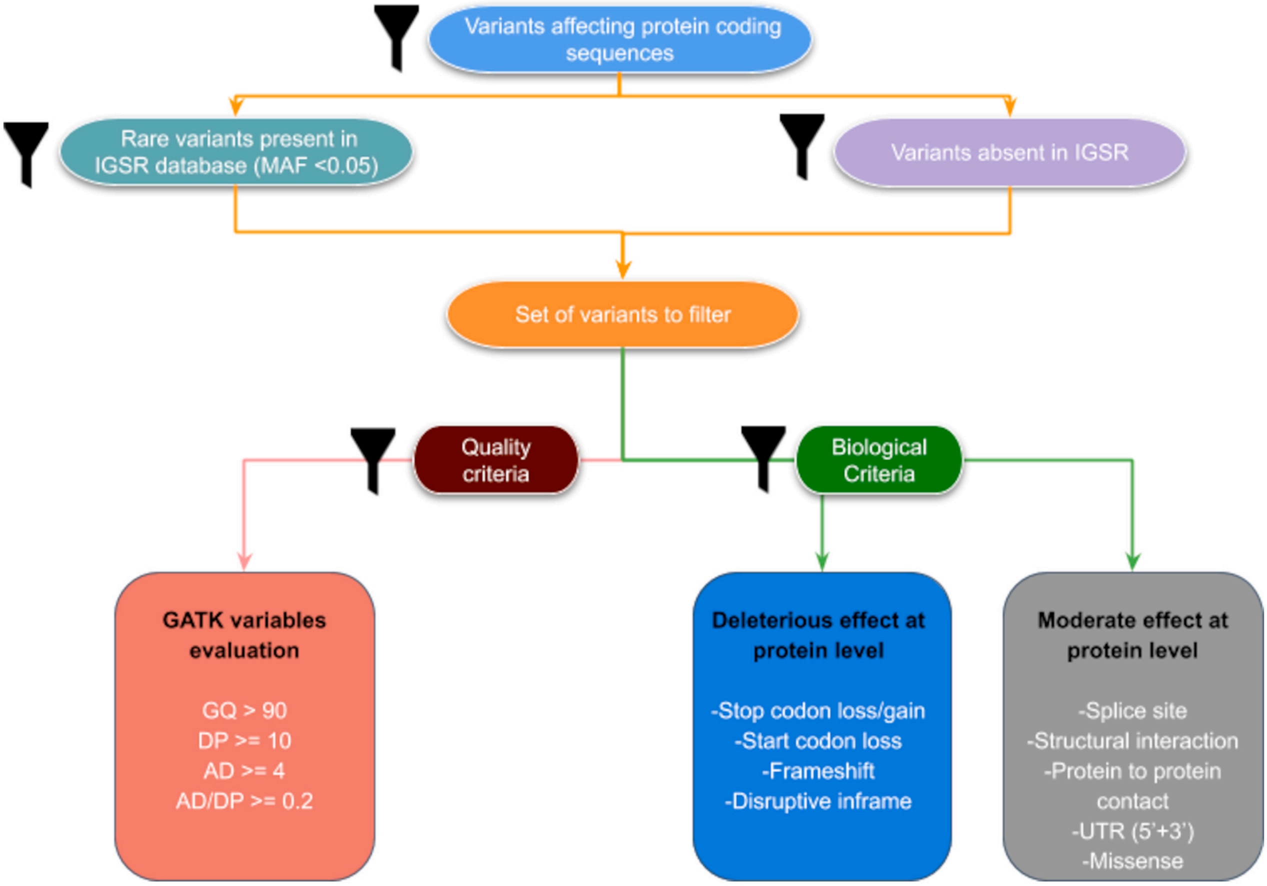

Figure S1: Pipeline for filtering genomic variants. A first filter was made to retain variants af-fecting protein coding sequences. From these, the International Genome Sample Resource (IGSR) database, which contains variants of >3000 healthy individuals, was consulted. Variants found in the database with a minor allele frequency (MAF) <0.05 were kept. Variants absent from the database also were retained. Two criteria were applied to this set of variants. Quality criteria values were based on confidence of a genotype attributed to a specific sample, or genotype quality (GQ); total amount of reads for a given position, or position depth (DP); and number of reads for the given variant detected in the sequencing, or allele depth (AD). In addition, AD should ac-count for ≥20% of position depth. Biological criteria were based on the predicted protein-level effect of the variant, which could range from deleterious to moderate.

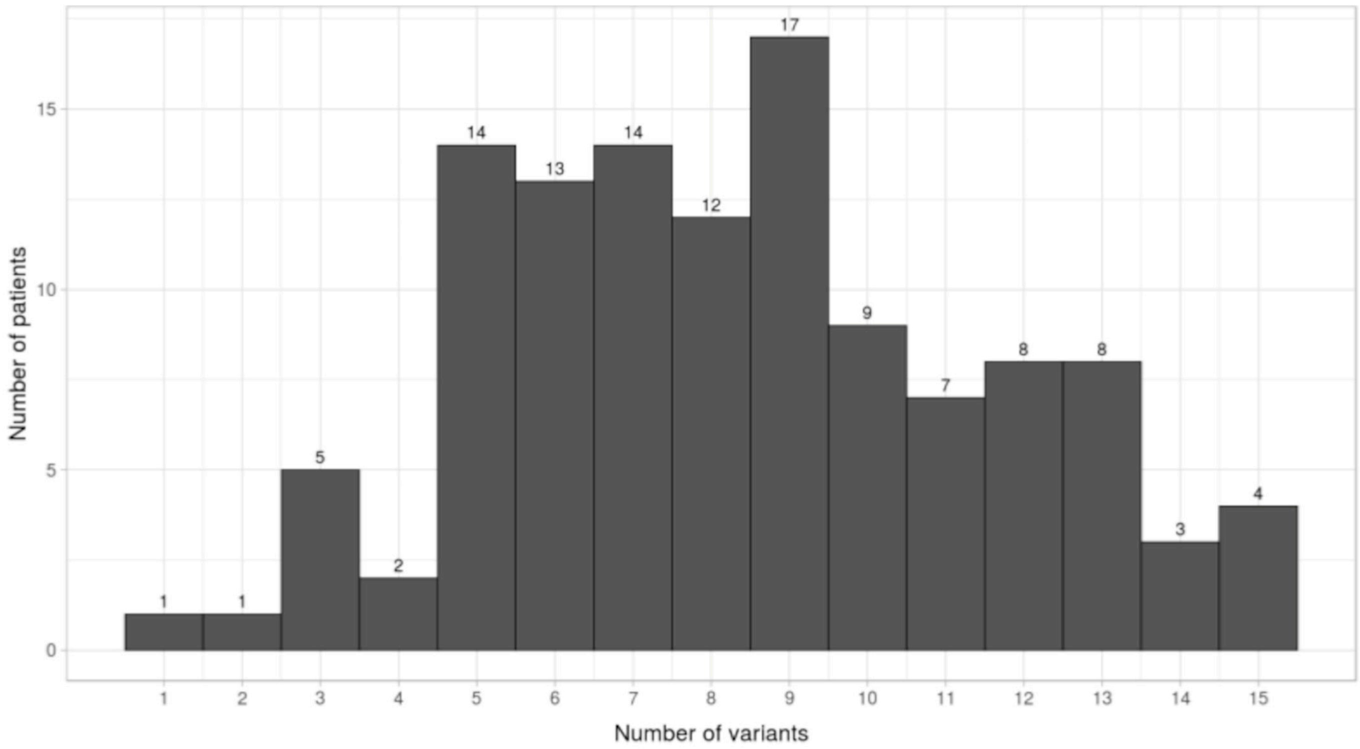

Figure S2: Frequency of ovarian failure variants accumulated in each patient. Number of patients is presented on the Y-axis, and number of presenting variants from the genomic profile of 66 variants associated with ovarian failure on the X-axis of the histogram. Most patients (17) shared 9 variants. The maximum number of shared variants was 15, seen in 4 patients.

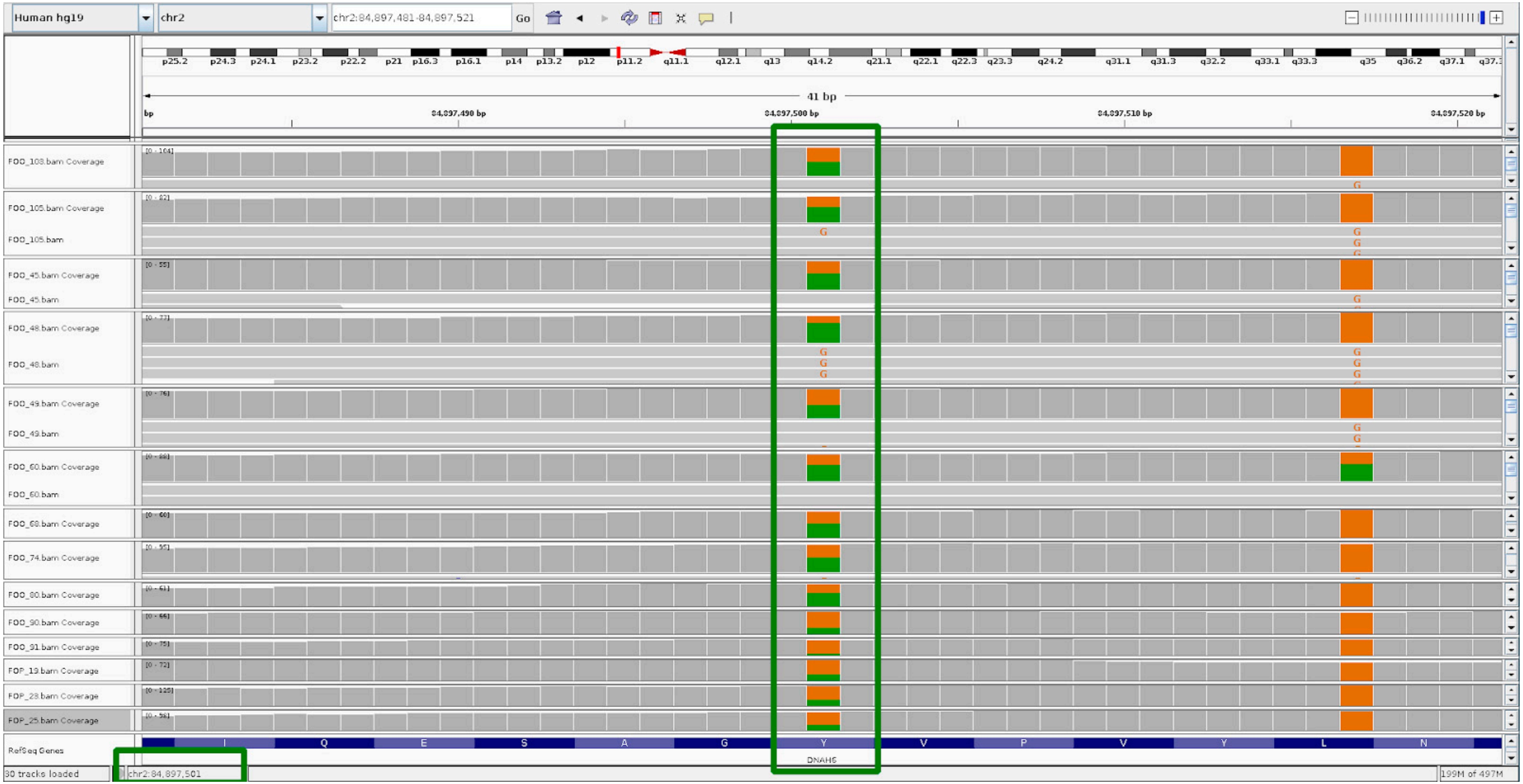
Human hg19
chr2
chr2:84,897,481-84,897,521
Go
41 bp
84,897,490 bp
84,897,500 bp
84,897,510 bp
84,897,520 bp
FOO_103.bam Coverage
FOO_105.bam Coverage
FOO_105.bam
FOO_45.bam Coverage
FOO_45.bam
FOO_48.bam Coverage
FOO_48.bam
FOO_49.bam Coverage
FOO_49.bam
FOO_60.bam Coverage
FOO_60.bam
FOO_68.bam Coverage
FOO_74.bam Coverage
FOO_80.bam Coverage
FOO_90.bam Coverage
FOO_91.bam Coverage
FOP_19.bam Coverage
FOP_23.bam Coverage
FOP_25.bam Coverage
RefSeq Genes
DNAH6
30 tracks loaded
chr2:84,897,501
199M of 497M

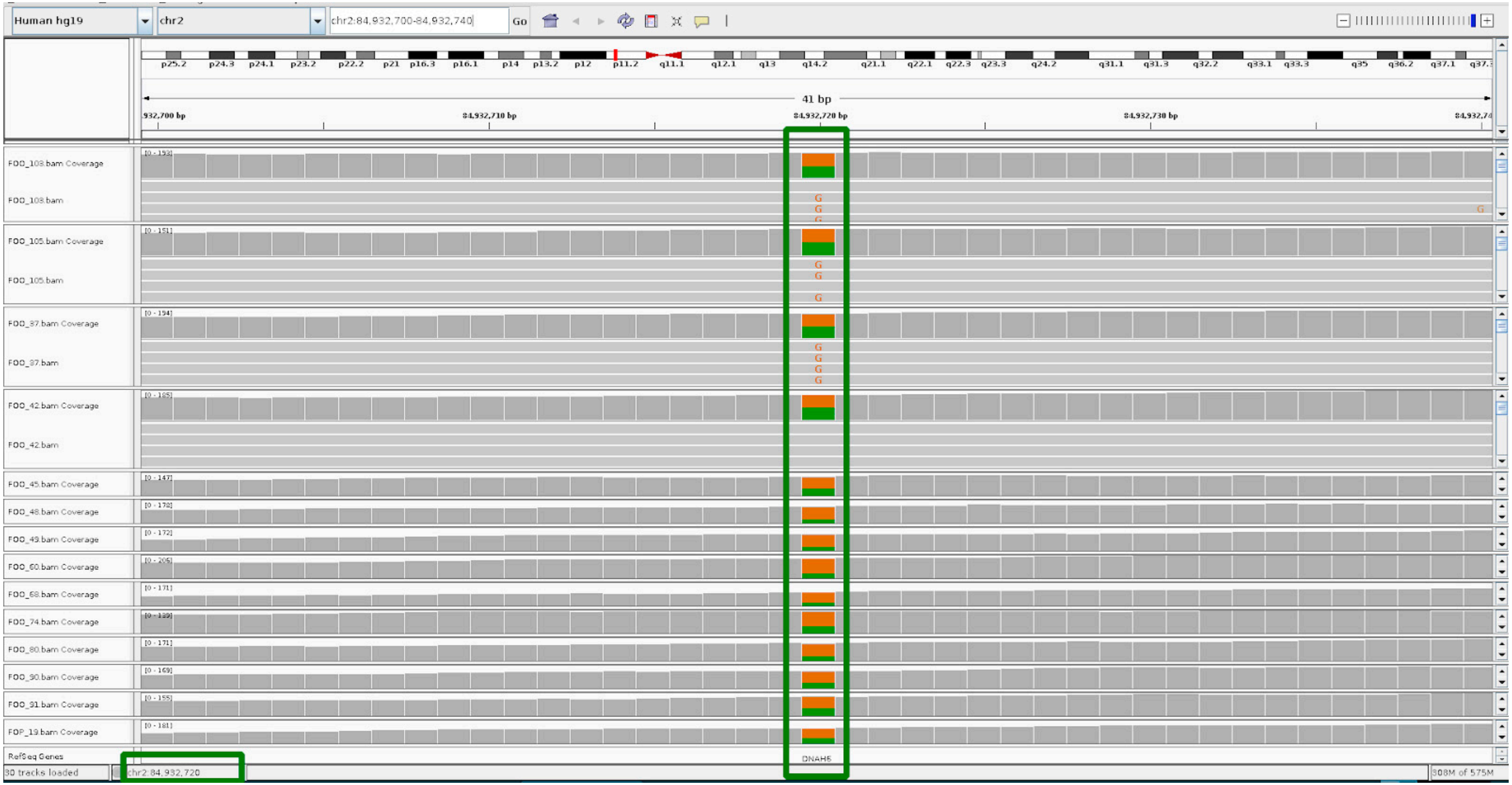
Human hg19
chr2
chr2:84,932,700-84,932,740
Go
41 bp
932,700 bp
84,932,710 bp
84,932,720 bp
84,932,730 bp
FOO_103.bam Coverage
FOO_103.bam
FOO_105.bam Coverage
FOO_105.bam
FOO_97.bam Coverage
FOO_97.bam
FOO_42.bam Coverage
FOO_42.bam
FOO_45.bam Coverage
FOO_48.bam Coverage
FOO_49.bam Coverage
FOO_60.bam Coverage
FOO_68.bam Coverage
FOO_74.bam Coverage
FOO_80.bam Coverage
FOO_90.bam Coverage
FOO_91.bam Coverage
FOP_19.bam Coverage
RefSeq Genes
DNAH5
30 tracks loaded
chr2:84,932,720
308M of 575M

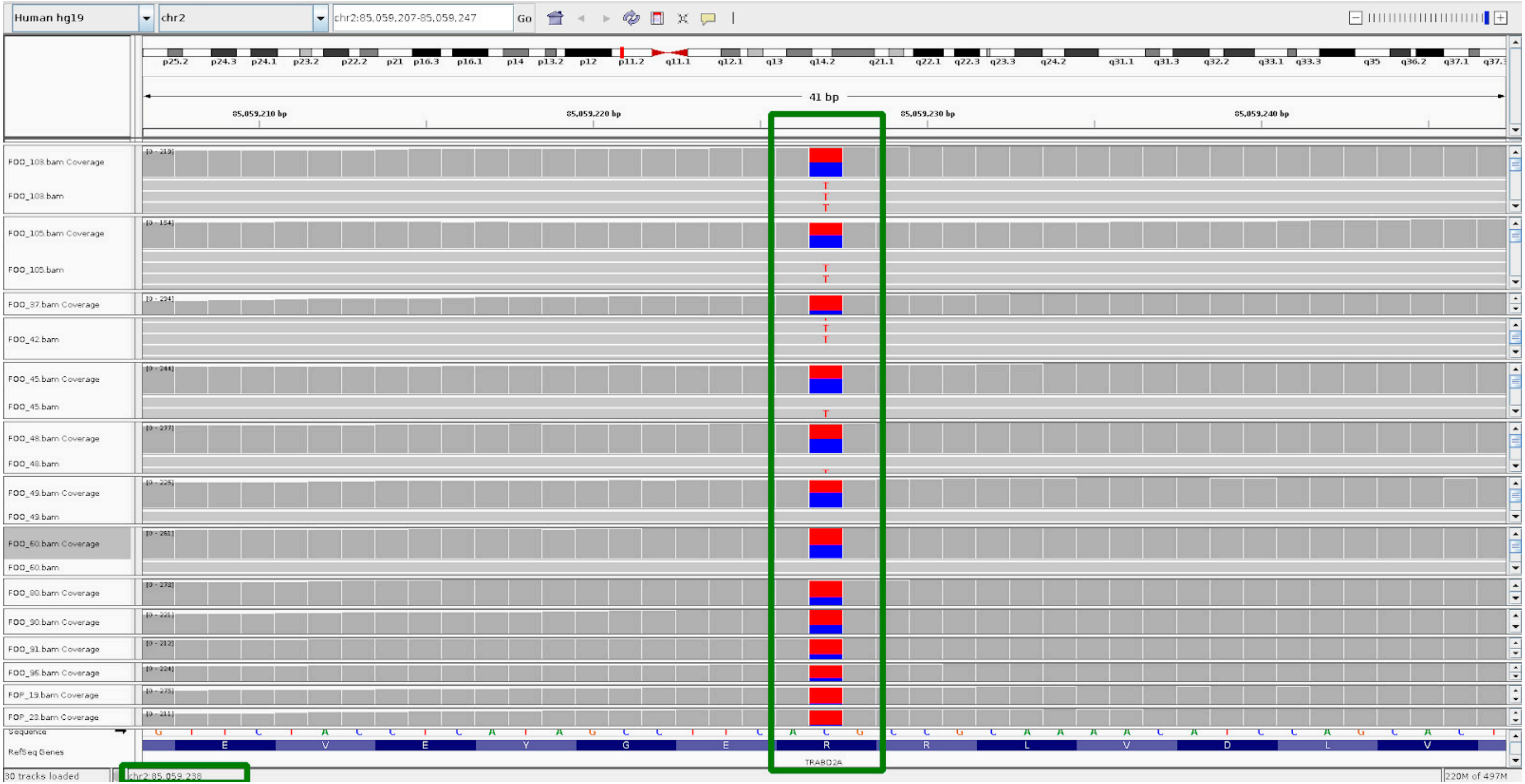

Figure S2: *DNAH6* and *TRABD2A* variants. Screenshots are shown for 3 variant s (2 for *DNAH6,* 1 for *TRABD2A*), highlighting the genome position (left corner, in green) and the presence of the variant in all corresponding case samples in the respective gene (highlighted in the centre of the picture, in green)

Chromosome & Position	Change at sequence & aa level	Type of change	Genes	Rs
1;1115503	c.289T>C , p.Cys97Arg	missense_variant	*TTLL10*	rs111751804
1;13183248	c.625A>T , p.Ser209Cys	missense_variant	*HNRNPCL2*	.
1;13183249	c.624G>T , p.Gln208His	missense_variant	*HNRNPCL2*	rs139566100
1;16245456	c.1431G>A ,	structural_interaction_variant	*SPEN*	rs146324760
1;17085995[1]	c.902C>G , p.Ala301Gly	missense_variant	*MST1L*	rs528252461
1;92554283	c.178G>A , p.Val60Ile	missense_variant	*BTBD8*	rs34856868
1;117560036	c.1553G>A , p.Arg518Gln	missense_variant	*CD101*	rs17235766
1;151867657	c.113C>G , p.Ser38Cys	missense_variant	*THEM4*	rs144257719
1;248458604	c.277G>A , p.Ala93Thr	missense_variant	*OR2T12*	rs143080755
2;26697422	c.3247G>C , p.Ala1083Pro	missense_variant	*OTOF*	rs80356574
2;26700099	c.2464C>T , p.Arg822Trp	missense_variant	*OTOF*	rs80356570
2;113147151	c.3371G>A , p.Gly1124Glu	missense_variant	*RGPD8*	.
3;30713794	c.1194G>A , p.Met398Ile	missense_variant	*TGFBR2*	rs35719192
3;48463799	c.1360A>C , p.Ser454Arg	missense_variant	*PLXNB1*	rs114964512
3;49548226	c.259A>G , p.Ile87Val	missense_variant	*DAG1*	rs116717961
3;58508273	c.1582C>G , p.Gln528Glu	missense_variant	*ACOX2*	rs143228600
3;68593660	c.*6G>C ,	3_prime_UTR_variant	*FAM19A1*	rs139431393
3;73673523	c.454C>T , p.His152Tyr	missense_variant	*PDZRN3*	rs112594802
3;75714788	c.445A>C , p.Ser149Arg	missense_variant	*FRG2C*	.
3;75787812	c.961dupA , p.Ser321fs	frameshift_variant	*ZNF717*	.
3;97595566	c.5528T>C , p.Ile1843Thr	missense_variant	*CRYBG3*	rs79662584
3;113765459	c.250C>A , p.His84Asn	missense_variant	*CCDC191*	rs138852073
3;183756692	c.1294C>A , p.Leu432Met	missense_variant	*HTR3D*	rs115389821
3;196807928	c.1998T>G , p.Asp666Glu	missense_variant	*DLG1*	rs35430440
3;197403779	c.2623G>A , p.Ala875Thr	missense_variant	*RUBCN*	rs183738683
4;175899001	c.2345_2398dupTGCCTTCCCAGAGTCATCCTCAGTTGACGCCTTCCCAGAGTCAACCTCCTGTGA , p.Met782_Val799dup	disruptive_inframe_insertion	*ADAM29*	.
4;187628419	c.2563G>A , p.Gly855Arg	missense_variant	*FAT1*	rs180820128
5;102537298	c.3797C>T , p.Thr1266Met	missense_variant	*PPIP5K2*	rs17155147
5;140568844	c.1952T>G , p.Leu651Arg	missense_variant	*PCDHB9*	rs17844538
5;140626900	c.1754T>C , p.Leu585Pro	missense_variant	*PCDHB15*	rs200613205
5;140712223	c.1972G>A , p.Val658Ile	missense_variant	*PCDHGA1*	rs144494457
5;147506593	c.3005A>G , p.His1002Arg	missense_variant	*SPINK5*	rs17705005
6;24429251	c.*9C>T ,	3_prime_UTR_variant	*GPLD1*	rs114756469
6;31378864	c.341A>G , p.Gln114Arg	missense_variant	*MICA*	rs41558312
6;32906586	c.212G>A , p.Ser71Asn	missense_variant	*HLA-DMB*	rs17617321
6;32906652	c.146A>T , p.Asp49Val	missense_variant	*HLA-DMB*	rs17617333
6;32906716	c.82A>G , p.Thr28Ala	missense_variant	*HLA-DMB*	rs17583782
6;36168628	c.529A>G , p.Ser177Gly	missense_variant	*BRPF3*	rs45504893
6;57246878	c.605T>C ,	protein_protein_contact	*PRIM2*	.
6;129571272	c.1798G>A , p.Gly600Arg	missense_variant	*LAMA2*	rs36044314
6;149721726	c.199A>G , p.Ile67Val	missense_variant	*SUMO4*	.

6;152784621	c.1964A>G , p.Gln655Arg	missense_variant	*SYNE1*	rs9397509
6;157099426	c.381_386dupGCAGCA , p.Gln128_Gln129dup	disruptive_inframe_insertion	*ARID1B*	.
7;33075573	c.9T>C , p.Asn3Asn	synonymous_variant	*NT5C3A*	rs17170223
7;115890509	c.661G>T , p.Ala221Ser	missense_variant	*TES*	rs142057704
7;138916697	c.467G>C , p.Arg156Pro	missense_variant&splice_region_variant	*UBN2*	rs141209093
7;139715645	c.1490C>A , p.Thr497Asn	missense_variant	*TBXAS1*	rs5763
8;24775457	c.2089G>C , p.Gly697Arg	missense_variant	*NEFM*	rs59726684
9;17394994	c.2542C>T , p.His848Tyr	missense_variant	*CNTLN*	rs144166918
9;32550912	c.58C>T , p.Pro20Ser	missense_variant	*TOPORS*	rs112527210
9;43737458	c.328C>T , p.Leu110Phe	missense_variant	*CNTNAP3B*	rs200069715
9;91616843	c.728G>A , p.Arg243Gln	missense_variant	*S1PR3*	rs34075341
9;140772477	c.92G>T , p.Gly31Val	missense_variant	*CACNA1B*	.
10;4872930	c.103G>A , p.Asp35Asn	missense_variant	*AKR1E2*	rs61745201
10;4889732	c.*10C>T ,	3_prime_UTR_variant	*AKR1E2*	rs74111762
10;46999484	c.604G>T , p.Gly202Trp	missense_variant	*GPRIN2*	.
10;47087501	c.718C>T , p.Arg240Cys	missense_variant	*NPY4R*	.
10;55582636	c.4871A>G , p.Asn1624Ser	missense_variant	*PCDH15*	rs111033362
10;70992634	c.341C>T , p.Thr114Met	missense_variant	*HKDC1*	rs35746503
10;81702210	c.367C>G , p.Leu123Val	missense_variant	*SFTPD*	rs17878336
10;82126541	c.410C>G , p.Pro137Arg	missense_variant	*DYDC2*	rs36027713
10;104162970	c.3062G>A , p.Arg1021Gln	missense_variant	*PSD*	.
10;105183348	c.2696T>C , p.Val899Ala	missense_variant	*PDCD11*	rs61751511
10;105362848	c.2040_2042dupCTC , p.Ser681dup	disruptive_inframe_insertion	*SH3PXD2A*	rs533143591
11;1017489	c.5312C>T , p.Pro1771Leu	missense_variant	*MUC6*	.
11;1017498	c.5303G>A , p.Ser1768Asn	missense_variant	*MUC6*	.
11;1017504	c.5297C>T , p.Thr1766Ile	missense_variant	*MUC6*	.
11;1018225	c.4572_4575delGCTA , p.Leu1525fs	frameshift_variant	*MUC6*	.
11;1018290	c.4511C>T , p.Pro1504Leu	missense_variant	*MUC6*	.
11;1651594	c.525_528delGTCC , p.Ser176fs	frameshift_variant	*KRTAP5-5*	.
11;1651599	c.530_555delGCTGCTGTAAGCCTTACTGCTGCCAG , p.Ser177fs	frameshift_variant	*KRTAP5-5*	.
11;7981261	c.1898G>A , p.Gly633Glu	missense_variant	*NLRP10*	rs116880092
11;18332440	c.325C>T , p.Arg109Cys	missense_variant	*HPS5*	rs202067617
11;56143424	c.325_326insCGGC , p.Ile109fs	frameshift_variant	*OR8U8*	rs12788990
11;124135140	c.418C>T , p.Leu140Phe	missense_variant	*OR8G5*	rs62622835
11;134237200	c.854C>T , p.Ser285Leu	missense_variant	*GLB1L2*	rs117422861
12;32977026	c.1759G>A , p.Val587Ile	missense_variant	*PKP2*	rs146102241
12;53491691	c.190C>T , p.Leu64Phe	missense_variant	*IGFBP6*	rs374700531
12;58217744	c.633A>G ,	structural_interaction_variant	*CTDSP2*	.
12;58220831	c.302G>C ,	structural_interaction_variant	*CTDSP2*	.
12;65672602	c.54C>T , p.Cys18Cys	synonymous_variant	*MSRB3*	rs149757878
12;96374622	c.1307G>A , p.Gly436Glu	missense_variant	*HAL*	rs181093412
12;122217559	c.481G>A , p.Ala161Thr	missense_variant	*RHOF*	rs34719836

13;25671369	c.1033G>T , p.Glu345*	stop_gained	*PABPC3*	.
13;50589928	c.300delA , p.Tyr101fs	frameshift_variant	*KCNRG*	rs576707522
14;44974189	c.2002G>T , p.Ala668Ser	missense_variant	*FSCB*	.
14;57755564	c.1435G>A , p.Ala479Thr	missense_variant	*AP5M1*	rs35759976
14;105419224	c.2564C>T , p.Ser855Leu	missense_variant	*AHNAK2*	rs117538559
15;22742777	c.1162G>A , p.Glu388Lys	missense_variant	*GOLGA6L1*	.
15;34678933	c.178G>A , p.Ala60Thr	missense_variant	*GOLGA8A*	.
15;49309158	c.1306A>G , p.Ile436Val	missense_variant	*SECISBP2L*	rs139762869
15;51693813	c.1051G>A , p.Asp351Asn	missense_variant	*GLDN*	rs35223886
15;51975608	c.374G>A , p.Ser125Asn	missense_variant	*SCG3*	rs2305710
15;69729049	c.1543T>C , p.Phe515Leu	missense_variant	*KIF23*	rs17310879
15;72462255	c.67G>A , p.Ala23Thr	missense_variant	*GRAMD2*	rs34815962
15;72511415	c.71C>T , p.Pro24Leu	missense_variant	*PKM*	rs3759901
15;90168171	c.4630C>T , p.Pro1544Ser	missense_variant	*TICRR*	rs144798110
16;733572	c.412G>A , p.Glu138Lys	missense_variant	*JMJD8*	rs187664088
16;2816519	c.5990G>A , p.Arg1997His	missense_variant	*SRRM2*	rs138447860
16;3406838	c.898G>A , p.Ala300Thr	missense_variant	*OR2C1*	rs62000975
16;81957106	c.2324A>G , p.Lys775Arg	missense_variant	*PLCG2*	rs142825971
16;84902483	c.880A>T , p.Met294Leu	missense_variant	*CRISPLD2*	rs72799568
16;88902199	c.692C>G ,	structural_interaction_variant	*GALNS*	rs34745339
17;5036748	c.287G>T , p.Arg96Leu	missense_variant	*USP6*	.
17;18542519	c.167A>C , p.Glu56Ala	missense_variant	*TBC1D28*	.
17;42745180	c.1901A>G , p.Tyr634Cys	missense_variant	*MEIOC*	rs77416189
17;59489707	c.371C>T , p.Ala124Val	missense_variant	*C17orf82*	rs77617620
18;52586545	c.746T>C , p.Val249Ala	missense_variant	*CCDC68*	rs34751112
19;847944	c.746G>A , p.Arg249His	missense_variant	*PRTN3*	rs150802678
19;4844790	c.850C>G , p.Gln284Glu	missense_variant	*PLIN3*	rs34070230
19;6156510	c.455A>G , p.Lys152Arg	missense_variant	*ACSBG2*	rs33937754
19;38160760	c.290C>T , p.Thr97Met	missense_variant	*ZNF781*	rs79684824
19;52918484	c.381delG , p.Ile129fs	frameshift_variant	*ZNF528*	rs562901913
21;30926024	c.2609C>T , p.Ala870Val	missense_variant&splice_region_variant	*GRIK1*	rs363503
22;35802661	c.539C>G , p.Thr180Ser	missense_variant	*MCM5*	rs2307340
X;76856021	c.5579A>G , p.Asn1860Ser	missense_variant	*ATRX*	rs45439799
9;17394994	c.2542C>T, p.His848Tyr	missense_variant	*CNTLN*	rs144166918
9;32550912	c.58C>T, p.Pro20Ser	missense_variant	*TOPORS*	rs112527210
9;43737458	c.328C>T, p.Leu110Phe	missense_variant	*CNTNAP3B*	rs200069715
9;91616843	c.728G>A, p.Arg243Gln	missense_variant	*S1PR3*	rs34075341
9;97063628	c.1791G>C, p.Gln597His	missense_variant	*ZNF169*	rs12350212
9;133768966	c.260G>A, p.Arg87His	missense_variant	*QRFP*	rs139997194
9;137642654	c.1588G>A, p.Gly530Ser	missense_variant	*COL5A1*	rs61735045
9;138516119	c.655C>T, p.Pro219Ser	missense_variant	*GLT6D1*	rs17040344
9;140509075	c.860A>C, p.Asn287Thr	missense_variant	*ARRDC1*	rs72765183
9;140772477	c.92G>T, p.Gly31Val	missense_variant	*CACNA1B*	.
10;4872930	c.103G>A, p.Asp35Asn	missense_variant	*AKR1E2*	rs61745201

10;4889732	c.*10C>T,	3_prime_UTR_variant	*AKR1E2*	rs74111762
10;46999484	c.604G>T, p.Gly202Trp	missense_variant	*GPRIN2*	.
10;47000217	c.1337G>A, p.Arg446His	missense_variant	*GPRIN2*	.
10;47087501	c.718C>T, p.Arg240Cys	missense_variant	*NPY4R*	.
10;55582636	c.4871A>G, p.Asn1624Ser	missense_variant	*PCDH15*	rs111033362
10;70992634	c.341C>T, p.Thr114Met	missense_variant	*HKDC1*	rs35746503
10;81702210	c.367C>G, p.Leu123Val	missense_variant	*SFTPD*	rs17878336
10;82126541	c.410C>G, p.Pro137Arg	missense_variant	*DYDC2*	rs36027713
10;95278683	c.1043G>A, p.Arg348Lys	missense_variant	*CEP55*	rs75139274
10;104162970	c.3062G>A, p.Arg1021Gln	missense_variant	*PSD*	.
10;105048373	c.1447A>C, p.Lys483Gln	missense_variant	*INA*	rs185636023
10;105183348	c.2696T>C, p.Val899Ala	missense_variant	*PDCD11*	rs61751511
10;105207196	c.685C>T, p.Gln229*	stop_gained	*CALHM2*	rs199696992
10;105362848	c.2040_2042dupCTC, p.Ser681dup	disruptive_inframe_insertion	*SH3PXD2A*	rs533143591
10;135086766	c.565C>T, p.Arg189Trp	missense_variant	*ADAM8*	rs45451297
10;135440214	c.33C>G, p.His11Gln	missense_variant	*FRG2B*	.
10;135440216	c.31C>T, p.His11Tyr	missense_variant	*FRG2B*	.
11;376331	c.1277T>A, p.Phe426Tyr	missense_variant	*B4GALNT4*	rs149045708
11;488541	c.796G>A, p.Val266Met	missense_variant	*PTDSS2*	rs34809643
11;1016800	c.6001C>T, p.Pro2001Ser	missense_variant	*MUC6*	.
11;1017466	c.5335A>T, p.Arg1779*	stop_gained	*MUC6*	.
11;1017489	c.5312C>T, p.Pro1771Leu	missense_variant	*MUC6*	.
11;1017498	c.5303G>A, p.Ser1768Asn	missense_variant	*MUC6*	.
11;1017504	c.5297C>T, p.Thr1766Ile	missense_variant	*MUC6*	.
11;1018225	c.4572_4575delGCTA, p.Leu1525fs	frameshift_variant	*MUC6*	.
11;1018279	c.4522G>A, p.Gly1508Arg	missense_variant	*MUC6*	.
11;1018290	c.4511C>T, p.Pro1504Leu	missense_variant	*MUC6*	.
11;1651594	c.525_528delGTCC, p.Ser176fs	frameshift_variant	*KRTAP5-5*	.
11;1651599	c.530_555delGCTGCTGTAAGCCTTACTGCTGCCAG, p.Ser177fs	frameshift_variant	*KRTAP5-5*	.
11;7981261	c.1898G>A, p.Gly633Glu	missense_variant	*NLRP10*	rs116880092
11;18267478	c.209C>T, p.Ala70Val	missense_variant	*SAA2-SAA4*	.
11;18332440	c.325C>T, p.Arg109Cys	missense_variant	*HPS5*	rs202067617
11;56143424	c.325_326insCGGC, p.Ile109fs	frameshift_variant	*OR8U8*	rs12788990
11;56468440	c.577G>T, p.Gly193Cys	missense_variant	*OR9G1*	.
11;60775227	c.314C>T, p.Pro105Leu	missense_variant	*CD6*	rs139918339
11;60785263	c.1615A>G, p.Thr539Ala	missense_variant	*CD6*	rs61755080
11;77815059	c.1316T>C, p.Ile439Thr	missense_variant	*ALG8*	rs17825668
11;108040484	c.2997C>A, p.Asn999Lys	missense_variant	*NPAT*	rs34052882
11;124135140	c.418C>T, p.Leu140Phe	missense_variant	*OR8G5*	rs62622835
11;124252750	c.490C>T, p.Leu164Phe	missense_variant	*OR8B2*	.
11;124253170	c.70C>T, p.Arg24Trp	missense_variant	*OR8B2*	.
11;124266697	c.550dupC, p.Leu184fs	frameshift_variant	*OR8B3*	rs201661436
11;124266906	c.342G>A, p.Met114Ile	missense_variant	*OR8B3*	.
11;134237200	c.854C>T, p.Ser285Leu	missense_variant	*GLB1L2*	rs117422861

12;11174476	c.695C>T, p.Thr232Ile	missense_variant	*TAS2R19*	.
12;11244731	c.98T>C, p.Ile33Thr	missense_variant	*TAS2R43*	rs201645619
12;11286323	c.521A>T, p.His174Leu	missense_variant	*TAS2R30*	rs200095034
12;32977026	c.1759G>A, p.Val587Ile	missense_variant	*PKP2*	rs146102241
12;50037071	c.2271G>A, p.Arg757Arg	synonymous_variant	*PRPF40B*	rs115544158
12;53491691	c.190C>T, p.Leu64Phe	missense_variant	*IGFBP6*	rs374700531
12;57132236	c.1126G>A, p.Val376Ile	missense_variant	*PRIM1*	rs143246089
12;58014864	c.379G>C, p.Gly127Arg	missense_variant	*SLC26A10*	rs190472657
12;58204145	c.748A>G, p.Ile250Val	missense_variant	*AVIL*	rs112269561
12;58217744	c.633A>G,	structural_interaction_variant	*CTDSP2*	.
12;58220831	c.302G>C,	structural_interaction_variant	*CTDSP2*	.
12;65672602	c.54C>T, p.Cys18Cys	synonymous_variant	*MSRB3*	rs149757878
12;96374622	c.1307G>A, p.Gly436Glu	missense_variant	*HAL*	rs181093412
12;103795425	c.107G>A, p.Ser36Asn	missense_variant	*C12orf42*	rs117838745
12;122217559	c.481G>A, p.Ala161Thr	missense_variant	*RHOF*	rs34719836
13;25671369	c.1033G>T, p.Glu345*	stop_gained	*PABPC3*	.
13;50589928	c.300delA, p.Tyr101fs	frameshift_variant	*KCNRG*	rs576707522
14;20296004	c.397C>T, p.Pro133Ser	missense_variant	*OR4N2*	.
14;21109745	c.106G>C, p.Val36Leu	missense_variant	*OR6S1*	rs45443395
14;21502110	c.338C>T, p.Ser113Phe	missense_variant	*RNASE13*	rs114504351
14;21502431	c.17C>T, p.Thr6Ile	missense_variant	*RNASE13*	rs113995906
14;44974189	c.2002G>T, p.Ala668Ser	missense_variant	*FSCB*	.
14;52508842	c.1806G>T, p.Glu602Asp	missense_variant	*NID2*	rs61747585
14;57755564	c.1435G>A, p.Ala479Thr	missense_variant	*AP5M1*	rs35759976
14;69341658	c.2663C>T, p.Pro888Leu	missense_variant	*ACTN1*	rs77499007
14;90650514	c.394C>T, p.Leu132Phe	missense_variant	*KCNK13*	rs55730840
14;105419224	c.2564C>T, p.Ser855Leu	missense_variant	*AHNAK2*	rs117538559
15;22742777	c.1162G>A, p.Glu388Lys	missense_variant	*GOLGA6L1*	.
15;34674034	c.1477G>A, p.Asp493Asn	missense_variant	*GOLGA8A*	.
15;34674048	c.1463C>T, p.Ala488Val	missense_variant	*GOLGA8A*	.
15;34678933	c.178G>A, p.Ala60Thr	missense_variant	*GOLGA8A*	.
15;41803754	c.680C>T, p.Pro227Leu	missense_variant	*LTK*	rs55739813
15;41822163	c.958G>A, p.Val320Met	missense_variant	*RPAP1*	rs61753586
15;42134416	c.1340C>T, p.Ala447Val	missense_variant	*JMJD7-PLA2G4B*	rs117260632
15;49309158	c.1306A>G, p.Ile436Val	missense_variant	*SECISBP2L*	rs139762869
15;51693813	c.1051G>A, p.Asp351Asn	missense_variant	*GLDN*	rs35223886
15;51975608	c.374G>A, p.Ser125Asn	missense_variant	*SCG3*	rs2305710
15;69729049	c.1543T>C, p.Phe515Leu	missense_variant	*KIF23*	rs17310879
15;72462255	c.67G>A, p.Ala23Thr	missense_variant	*GRAMD2*	rs34815962
15;72511415	c.71C>T, p.Pro24Leu	missense_variant	*PKM*	rs3759901
15;78558584	c.1_2delAT, p.Met1fs	frameshift_variant&start_lost	*DNAJA4*	rs142025971
15;90168171	c.4630C>T, p.Pro1544Ser	missense_variant	*TICRR*	rs144798110
15;99670847	c.2279C>T, p.Pro760Leu	missense_variant	*SYNM*	rs3743247

15;99671765	c.3197G>C, p.Arg1066Pro	missense_variant	*SYNM*	rs5030698
16;733572	c.412G>A, p.Glu138Lys	missense_variant	*JMJD8*	rs187664088
16;2816519	c.5990G>A, p.Arg1997His	missense_variant	*SRRM2*	rs138447860
16;3406838	c.898G>A, p.Ala300Thr	missense_variant	*OR2C1*	rs62000975
16;20802007	c.1323C>G, p.Tyr441*	stop_gained	*ACSM3*	rs34381224
16;81922813	c.802C>T, p.Arg268Trp	missense_variant	*PLCG2*	rs1143687
16;81957106	c.2324A>G, p.Lys775Arg	missense_variant	*PLCG2*	rs142825971
16;84902483	c.880A>T, p.Met294Leu	missense_variant	*CRISPLD2*	rs72799568
16;88902199	c.692C>G,	structural_interaction_variant	*GALNS*	rs34745339
17;1840677	c.439G>A, p.Gly147Ser	missense_variant	*RTN4RL1*	rs181444163
17;1943880	c.1003C>G, p.Leu335Val	missense_variant	*DPH1*	rs35394823
17;4060383	c.*7G>A,	3_prime_UTR_variant	*CYB5D2*	rs76481447
17;5036748	c.287G>T, p.Arg96Leu	missense_variant	*USP6*	.
17;18542519	c.167A>C, p.Glu56Ala	missense_variant	*TBC1D28*	.
17;30351758	c.1708C>A, p.Leu570Ile	missense_variant	*LRRC37B*	.
17;42745180	c.1901A>G, p.Tyr634Cys	missense_variant	*MEIOC*	rs77416189
17;59489707	c.371C>T, p.Ala124Val	missense_variant	*C17orf82*	rs77617620
17;62499163	c.864G>A,	structural_interaction_variant	*DDX5*	rs56025355
17;79205672	c.676C>T, p.Arg226Trp	missense_variant	*TEPSIN*	rs61745844
18;44057673	c.6398G>A, p.Arg2133His	missense_variant	*LOXHD1*	rs74316327
18;51880889	c.55C>T, p.Arg19*	stop_gained	*STARD6*	rs17292725
18;52586545	c.746T>C, p.Val249Ala	missense_variant	*CCDC68*	rs34751112
18;72913537	c.968G>T, p.Cys323Phe	missense_variant	*ZADH2*	rs17056661
19;847944	c.746G>A, p.Arg249His	missense_variant	*PRTN3*	rs150802678
19;4844790	c.850C>G, p.Gln284Glu	missense_variant	*PLIN3*	rs34070230
19;6156510	c.455A>G, p.Lys152Arg	missense_variant	*ACSBG2*	rs33937754
19;12186732	c.797A>G, p.Asn266Ser	missense_variant	*ZNF844*	rs76842919
19;12541250	c.1736A>G, p.Glu579Gly	missense_variant	*ZNF443*	rs45531232
19;12541541	c.1444_1445insCA, p.Gly482fs	frameshift_variant	*ZNF443*	.
19;12551729	c.-1A>T,	5_prime_UTR_variant	*ZNF443*	rs62110751
19;38160760	c.290C>T, p.Thr97Met	missense_variant	*ZNF781*	rs79684824
19;39972581	c.476G>C, p.Gly159Ala	missense_variant	*TIMM50*	rs114065046
19;45316704	c.611T>A, p.Met204Lys	missense_variant	*BCAM*	rs28399656
19;46997016	c.1707G>A, p.Arg569Arg	synonymous_variant	*PNMAL2*	rs147338403
19;52520372	c.479G>A, p.Gly160Glu	missense_variant	*ZNF614*	rs45596739
19;52918484	c.381delG, p.Ile129fs	frameshift_variant	*ZNF528*	rs562901913
19;55284962	c.248C>G, p.Ala83Gly	missense_variant	*KIR2DL1*	.
19;55320293	c.661C>T, p.Pro221Ser	missense_variant	*KIR2DL4*	.
19;55320305	c.674_675delGG, p.Trp225fs	frameshift_variant	*KIR2DL4*	.
19;55320308	c.676_677insAT, p.Pro226fs	frameshift_variant	*KIR2DL4*	.
19;55377265	c.1006T>C, p.Cys336Arg	missense_variant	*KIR3DL2*	.
19;55377325	c.1066C>T, p.Leu356Phe	missense_variant	*KIR3DL2*	.
19;55377340	c.1081T>C, p.Tyr361His	missense_variant	*KIR3DL2*	.
19;55377343	c.1084C>T, p.Arg362Cys	missense_variant	*KIR3DL2*	.
19;55377348	c.1089G>C, p.Trp363Cys	missense_variant	*KIR3DL2*	.

20;746197	c.222C>G, p.Ile74Met	missense_variant	*SLC52A3*	rs35655964
20;30449420	c.185T>G, p.Leu62Trp	missense_variant	*DUSP15*	rs184251613
20;35675512	c.1549C>T,	structural_interaction_variant	*RBL1*	rs149999468
20;36572456	c.416C>T, p.Thr139Ile	missense_variant	*VSTM2L*	rs139749355
20;61598731	c.1190C>T, p.Thr397Met	missense_variant	*SLC17A9*	rs7271712
21;30926024	c.2609C>T, p.Ala870Val	missense_variant&splice_region_variant	*GRIK1*	rs363503
21;47545369	c.1817-3dupC,	splice_acceptor_variant&intron_variant	*COL6A2*	.
22;29130458	c.252A>G, p.Glu84Glu	synonymous_variant	*CHEK2*	rs1805129
22;35802661	c.539C>G, p.Thr180Ser	missense_variant	*MCM5*	rs2307340
22;51063610	c.1493G>A, p.Arg498His	missense_variant	*ARSA*	rs6151428
X;48924896	c.1246C>A, p.Leu416Met	missense_variant	*CCDC120*	rs55654138
X;57162895	c.136C>T, p.Arg46Cys	missense_variant	*SPIN2A*	rs202201318
X;76856021	c.5579A>G, p.Asn1860Ser	missense_variant	*ATRX*	rs45439799
X;138686878	c.1933C>T, p.His645Tyr	missense_variant	*MCF2*	rs61751333

Supplementary Table 1: **Significant variants found after employing contrast of proportions**. One hundred sixteen significant variants were identified using the Fisher test ($p < 0.01$). Chromosome, position, change at the sequence and amino acid level, type of change, genes affected, and dbSNP identifier, if known, are shown. 1 = Significant variant after adjusting p-values for FDR (adj. < 0.05).

Chromosome and position	Change at sequence and aa level	Rs	Type of change	Amino acid class, polarity, and charge change	Gene	Function	N cases affected	Coverage	Accession number
1;17086085	c.811dupG, p.Ala271fs	.	Frameshift variant	Aliphatic nonpolar neutral > frameshift	*MST1L*	Proteolysis	19	1.343	NM_001271733.1
1;89525966	c.232A>G, p.Ile78Val	rs1048401	Structural interaction variant	Aliphatic nonpolar neutral > aliphatic nonpolar neutral	*GBP1*	Immune system process	12	236	NM_002053.2
1;207857222	c.383G>A, p.Arg128Gln	rs41314532	Missense variant	Basic polar positive > amide polar neutral	*CR1L*	Regulation of complement activation	12	315	NM_175710.1
1;244641216	c.197C>A, p.Thr66Lys	rs11586356	Missense variant	Hydroxylic polar neutral > basic polar positive	*CATSPERE*	Sperm cell hyperactivation	17	151	NM_001130957.1
2;21224853	c.13441G>A, p.Ala4481Thr	rs1801695	Missense variant	Aliphatic nonpolar neutral > hydroxylic polar neutral	*APOB*	Toll-like receptor signaling pathway	13	212	NM_000384.2
2;69650730	c.286C>T, p.Arg96Cys	rs74637005	Missense variant	Basic polar positive > sulfuric nonpolar neutral	*NFU1*	Iron-sulfur cluster assembly	13	142	NM_001002755.2
2;84897501[2]	c.6356A>G, p.Tyr2119Cys	rs17025409	Missense variant	Aromatic polar neutral > sulfuric nonpolar neutral	*DNAH6*	Microtubule activity	17	146	NM_001370.1
2;84932720[2]	c.8576A>G, p.Lys2859Arg	rs61750773	Missense variant	Basic polar positive > basic polar positive	*DNAH6*	Microtubule activity	19	146	NM_001370.1
2;85059227[2]	c.1034G>A, p.Arg345His	rs61744273	Missense variant	Basic polar positive > basic aromatic polar positive-neutral	*TRABD2A*	Negative regulation of WNT signaling pathway	18	287	NM_001277053.1
2;132237927	c.661C>A, p.Arg221Ser	.	Missense variant	Basic polar positive > hydroxylic polar neutral	*TUBA3D*	Cytoskeleton organisation	17	551	NM_080386.3
2;176829117	c.674C>G, p.Thr225Ser	rs34897061	Missense variant	Hydroxylic polar neutral > hydroxylic polar neutral	*LNPK*	Endoplasmic reticulum organisation	13	121	NM_001305008.1
2;207569623	c.427T>G, p.Ser143Ala	rs116768218	Missense variant	Hydroxylic polar neutral > aliphatic nonpolar neutral	*DYTN*	Calcium ion binding	16	207	NM_001093730.1
3;46945000	c.1636G>A, p.Glu546Lys	rs77048718	Missense variant	Acid acidic polar negative > basic polar positive	*PTH1R*	Phospholipase C-activating G protein	15	251	NM_000316.2

3;196921360	c.419A>G, p.Lys140Arg	rs1802668	Missense variant	Basic polar positive > basic polar positive	*DLG1*	MAPK cascade	13	135	NM_004087.2
4;6610889	c.1870G>A, p.Gly624Arg	rs61733402	Missense variant	Aliphatic nonpolar neutral > basic polar positive	*MAN2B2*	Oligosaccharide catabolic process	19	338	NM_015274.2
4;78830434	c.685G>C, p.Asp229His	rs1801350	Missense variant	Acid acidic polar negative > basic aromatic polar positive-neutral	*MRPL1*	Mitochondrial translational elongation/termination	15	142	NM_020236.3
4;82355804	c.1189G>A, p.Val397Ile	rs34211143	Missense variant	Aliphatic nonpolar neutral > aliphatic nonpolar neutral	*RASGEF1B*	Small GTPase mediated signal transduction	13	146	NM_152545.2
4;99273704	c.316G>A, p.Val106Met	rs61758810	Missense variant	Aliphatic nonpolar neutral > sulfuric nonpolar neutral	*RAP1GDS1*	Positive regulation of GTPase activity	14	157	NM_001100426.1
4;148560193	c.2240G>A, p.Cys747Tyr	rs11557361	Missense variant	Sulfuric nonpolar neutral > aromatic polar neutral	*PRMT9*	mRNA processing	12	167	NM_138364.3
4;187122332	c.823G>A, p.Glu275Lys	rs34745240	Missense variant	Acid acidic polar negative > basic polar positive	*CYP4V2*	Retinoid metabolic process	12	205	NM_207352.3
5;79950724[3]	c.181G>C, p.Ala60Pro	rs2001675	Missense variant	Aliphatic nonpolar neutral > cyclic nonpolar neutral	*MSH3*	DNA repair	15	145	NM_002439.4
6;27279774	c.176G>A, p.Arg59Gln	rs17688097	Missense variant	Basic polar positive > amide polar neutral	*POM121L2*	Protein import into nucleus	12	271	NM_033482.3
6;32489855	c.197A>T, p.Asp66Val	.	Missense variant	Acid acidic polar negative > aliphatic nonpolar neutral	*HLA-DRB5*	Interferon-gamma-mediated signaling pathway	14	266	NM_002125.3
6;36168628[1]	c.529A>G, p.Ser177Gly	rs45504893	Missense variant	Hydroxylic polar neutral > aliphatic nonpolar neutral	*BRPF3*	Chromatin organisation	22	320	NM_015695.2
6;143929450	c.4G>A, p.Asp2Asn	rs41285023	Missense variant	Acid acidic polar negative > amide polar neutral	*PHACTR2*	Platelet degranulation	13	194	NM_001100166.1
6;151673074	c.3548C>G, p.Pro1183Arg	rs41289375	Missense variant	Cyclic nonpolar neutral > basic polar positive	*AKAP12*	Regulation of protein kinase A signaling	16	216	NM_005100.3
7;11022560	c.674C>G, p.Ser225Cys	rs61753124	Missense variant	Hydroxylic polar neutral > sulfuric nonpolar neutral	*PHF14*	Negative regulation of	12	171	NM_014660.3

						transcription			
7;12675690	c.1340G>A, p.Arg447Gln	rs35385652	Missense variant	Basic polar positive > amide polar neutral	*SCIN*	Negative regulation of cell proliferation	15	153	NM_001112706.2
7;12675713	c.1363T>C, p.Phe455Leu	rs17166250	Missense variant	Aromatic nonpolar neutral > aliphatic nonpolar neutral	*SCIN*	Negative regulation of cell proliferation	15	153	NM_001112706.2
9;69391207[4]	c.715G>A, p.Ala239Thr	.	Missense variant	Aliphatic nonpolar neutral > hydroxylic polar neutral	*ANKRD20A4*	Unknown	13	125	NM_001098805.1
9;97063628	c.1791G>C, p.Gln597His	rs12350212	Missense variant	Amide polar neutral > basic aromatic polar positive-neutral	*ZNF169*	Regulation of transcription	19	359	NM_003448.2
9;138516119	c.655C>T, p.Pro219Ser	rs17040344	Missense variant	Cyclic nonpolar neutral > hydroxylic polar neutral	*GLT6D1*	Lipid glycosylation	18	205	NM_182974.2
10;16932490	c.8635C>A, p.Leu2879Ile	rs1801238	Missense variant	Aliphatic nonpolar neutral > aliphatic nonpolar neutral	*CUBN*	Cobalamin transport	12	155	NM_001081.3
10;17243638	c.-5C>G	rs11254458	5′ UTR variant	-	*TRDMT1*	tRNA modification	12	120	NM_004412.6
11;1017471[2]	c.5330G>A, p.Gly1777Asp	.	Missense variant	Aliphatic nonpolar neutral > acid acidic polar negative	*MUC6*	Cytoprotection of epithelial surfaces	13	448	NM_005961.2
11;1017504[1,2]	c.5297C>T, p.Thr1766Ile	.	Missense variant	Hydroxylic polar neutral > aliphatic nonpolar neutral	*MUC6*	Cytoprotection of epithelial surfaces	31	448	NM_005961.2
11;102248377	c.1517C>T, p.Ala506Val	rs34510872	Missense variant	Aliphatic nonpolar neutral > aliphatic nonpolar neutral	*BIRC2*	Protein polyubiquitination	14	163	NM_001166.4
11;107328544	c.-2C>T	rs2276271	Premature start codon gain variant	-	*CWF19L2*	mRNA splicing, via spliceosome	15	124	NM_152434.2
14;20002224	c.1197+1G>A	.	Splice donor variant	-	*POTEM*	Unknown	15	301	NM_001145442.1
14;57755564[1]	c.1435G>A, p.Ala479Thr	rs35759976	Missense variant	Aliphatic nonpolar neutral > hydroxylic polar neutral	*AP5M1*	Apoptosis	22	179	NM_018229.3
15;41803754	c.680C>T, p.Pro227Leu	rs55739813	Missense variant	Cyclic nonpolar neutral > aliphatic nonpolar neutral	*LTK*	Signal transduction	17	225	NM_002344.5

15;41822163	c.958G>A, p.Val320Met	rs61753586	Missense variant	Aliphatic nonpolar neutral > sulfuric nonpolar neutral	*RPAP1*	Transcription by RNA polymerase II	18	251	NM_015540.3
16;2263836	c.859G>A, p.Gly287Arg	rs116977380	Missense variant	Aliphatic nonpolar neutral > basic polar positive	*PGP*	Dephosphorylation	13	138	NM_001042371.2
16;15818842	c.3887T>C, p.Val1296Ala	rs16967510	Missense variant	Aliphatic nonpolar neutral > aliphatic nonpolar neutral	*MYH11*	Elastic fiber assembly	15	296	NM_001040114.1
16;25239809[3,2]	c.782G>A, p.Arg261Gln	rs1118401 56	Missense variant	Basic polar positive > amide polar neutral	*AQP8*	Cellular response to cAMP	13	265	NM_001169.2
16;30393147	c.380G>T, p.Gly127Val	rs34518080	Missense variant	Aliphatic nonpolar neutral > aliphatic nonpolar neutral	*SEPTIN1*	Cytoskeleton-dependent cytokinesis	12	288	NM_052838.4
16;57732012	c.151G>A, p.Asp51Asn	rs55645458	Missense variant	Acid acidic polar negative > amide polar neutral	*DRC7*	Flagellated sperm motility	13	314	NM_001289162.1
16;84902483[1]	c.880A>T, p.Met294Leu	rs72799568	Missense variant	Sulfuric nonpolar neutral > aliphatic nonpolar neutral	*CRISPLD2*	Extracellular matrix assembly	21	247	NM_031476.3
16;88902199[1]	c.692C>G, p.Ala237Gly	rs34745339	Structural interaction variant, missense variant	Aliphatic nonpolar neutral > aliphatic nonpolar neutral	*GALNS*	Degradation of glycosaminoglycans	20	214	NM_000512.4
17;10366204	c.986A>G, p.Gln329Arg	rs35984286	Missense variant	Amide polar neutral > basic polar positive	*MYH4*	ATP metabolic process	13	197	NM_017533.2
17;34798001	c.1135G>A, p.Gly379Arg	.	Missense variant	Aliphatic nonpolar neutral > basic polar positive	*TBC1D3B*	Intracellular protein transport	12	251	NM_001001417.6
17;47300081	c.*4C>G	rs2233374	3′ UTR variant	-	*ABI3*	Regulation of cell migration	12	215	NM_016428.2
17;66972055	c.*1A>C	rs72851722	3′ UTR variant	-	*ABCA9*	Lipid transport	12	162	NM_080283.3
17;67149972	c.3965G>A, p.Arg1322Gln	rs72852601	Missense variant	Basic polar positive > amide polar neutral	*ABCA10*	Lipid transport	12	126	NM_080282.3
17;71232990[2]	c.1369C>A, p.Arg457Ser	rs61729639	Missense variant	Basic polar positive > hydroxylic polar neutral	*SPEP1*	Unknown	14	180	NM_001288771.1
18;658147	c.101G>T, p.Cys34Phe	rs182344736	Missense variant	Sulfuric nonpolar neutral > aromatic nonpolar neutral	*TYMSOS*	Unknown	12	654	NM_001012716.2
19;10671894	c.554A>C, p.Glu185Ala	rs11545166	Missense variant	Acid acidic polar negative > aliphatic nonpolar neutral	*KRI1*	Endonucleolytic cleavage	13	327	NM_023008.3

19;41356246	c.86G>A, p.Ser29Asn	rs28399435	Missense variant	Hydroxylic polar neutral > amide polar neutral	*CYP2A6*	Steroid metabolic process	12	559	NM_000762.5
19;49102599	c.1034C>T, p.Pro345Leu	rs17842463	Missense variant	Cyclic nonpolar neutral > aliphatic nonpolar neutral	*SULT2B1*	Negative regulation of cell proliferation	13	277	NM_177973.1
19;52520372	c.479G>A, p.Gly160Glu	rs45596739	Missense variant	Aliphatic nonpolar neutral > acid acidic polar negative	*ZNF614*	Regulation of transcription	17	187	NM_025040.3
22;17450929[2]	c.841G>A, p.Ala281Thr	rs61741409	Missense variant	Aliphatic nonpolar neutral > hydroxylic polar neutral	*GAB4*	Unknown	14	236	NM_001037814.1
22;17450952[2]	c.818T>C, p.Leu273Pro	rs11703655	Missense variant	Aliphatic nonpolar neutral > cyclic nonpolar neutral	*GAB4*	Unknown	14	236	NM_001037814.1
22;25024326[3]	c.1534G>A, p.Val512Ile	.	Structural interaction variant, missense variant	Aliphatic nonpolar neutral > aliphatic nonpolar neutral	*GGT1*	Proteolysis	14	112	NM_013430.2
22;35802661[1]	c.539C>G, p.Thr180Ser	rs2307340	Missense variant	Hydroxylic polar neutral > hydroxylic polar neutral	*MCM5*	DNA replication initiation	20	309	NM_006739.3
X;129306052	c.16C>G, p.Leu6Val	rs11542874	Missense variant	Aliphatic nonpolar neutral > aliphatic nonpolar neutral	*RAB33A*	Rab protein signal transduction	12	418	NM_004794.2
X;151123384	c.1310C>T, p.Pro437Leu	rs45439991	Missense variant	Cyclic nonpolar neutral > aliphatic nonpolar neutral	*GABRE*	Signal transduction	15	254	NM_004961.3

Supplementary Table S2: Novel sixty-six variants predictive of ovarian failure. Chromosomal and genomic positions of the variants, as well as bases and amino acid changes at the indicated number in the sequences, dbSNP IDs (if known), type of changes, amino acid class, polarity and charge changes, genes affected, number of cases affected by the variant, coverage and accession numbers. Variants were present in at least 10% of cases (12 women out of 118) and no controls. Gene Ontology and Genecards databases were consulted to annotate gene function. [1]Top 6 variants shared by >20 cases. [2]Top variants valued by random forest algorithm to stratify the population. [3]The three variants found affecting genes already related to ovarian failure in the literature. [4]Variant absent in International Genome Sample Resource, dbSNP, and GnomAD databases.
